# Brain fingerprinting field studies comparing P300-MERMER and P300 brainwave responses in the detection of concealed information

**DOI:** 10.1007/s11571-012-9230-0

**Published:** 2012-12-05

**Authors:** Lawrence A. Farwell, Drew C. Richardson, Graham M. Richardson

**Affiliations:** 1 Government Works, Inc., Brainwave Science, 257 Turnpike Road, Southborough, MA 01772 USA; 2 Present Address: Federal Bureau of Investigation (FBI) Laboratory, 314 High Meadow Lane, Greenville, VA 24440 USA; 3 Department of Cell and Developmental Biology, Vanderbilt University, MRB III Laboratory U 3200, 465 21st Ave. South, Nashville, TN 37232 USA

**Keywords:** Brain fingerprinting, P300-MERMER, P300, Event-related potential, Detection of concealed information, MERMER

## Abstract

Brain fingerprinting detects concealed information stored in the brain by measuring brainwave responses. We compared P300 and P300-MERMER event-related brain potentials for error rate/accuracy and statistical confidence in four field/real-life studies. 76 tests detected presence or absence of information regarding (1) real-life events including felony crimes; (2) real crimes with substantial consequences (either a judicial outcome, i.e., evidence admitted in court, or a $100,000 reward for beating the test); (3) knowledge unique to FBI agents; and (4) knowledge unique to explosives (EOD/IED) experts. With both P300 and P300-MERMER, error rate was 0 %: determinations were 100 % accurate, no false negatives or false positives; also no indeterminates. Countermeasures had no effect. Median statistical confidence for determinations was 99.9 % with P300-MERMER and 99.6 % with P300. Brain fingerprinting methods and scientific standards for laboratory and field applications are discussed. Major differences in methods that produce different results are identified. Markedly different methods in other studies have produced over 10 times higher error rates and markedly lower statistical confidences than those of these, our previous studies, and independent replications. Data support the hypothesis that accuracy, reliability, and validity depend on following the brain fingerprinting scientific standards outlined herein.

## Introduction and background

This paper reports four field/real-life studies using event-related potentials in the detection of concealed information. A primary purpose of these studies was to test the effectiveness of certain very specific methods for using brain responses in the detection of concealed information. We tested these specific methods in two types of field/real-life tests in detecting concealed information obtained in the course of real-life events. Two studies were specific issue tests. They used event-related brain potentials to detect concealed information regarding specific incidents in the lives of subjects, including major crimes with life-changing judicial outcomes. Two studies were specific screening or focused screening tests.^1^ They used event-related potentials to detect knowledge related to a particular kind of training or expertise, specifically knowledge characteristic of FBI agents and knowledge characteristic of explosives experts or bomb makers.

Another major purpose of the research reported herein is to identify the scientific principles and specific methods required to obtain valid and reliable results, an extremely low error rate, and high statistical confidence for all determinations made. We also have sought to identify the specific principles and methods required to obtain resistance to countermeasures and to minimize indeterminate outcomes while maintaining an extremely low error rate.

Due to overriding security concerns, it has not previously been possible to publish details of some of our research at the CIA, the FBI, the U.S. Navy, and elsewhere. These concerns have now been resolved, and this research can now be published. This is the fourth in a series of six recent peer-reviewed articles to be published, beginning with Farwell (2011a, 2012, 2013). We have also published two new patents (Farwell 2007, 2010) and other papers (e.g., Farwell 2011b). We hope through these publications to address some of the major issues that have arisen in the field since our original publications (Farwell and Donchin 1991; Farwell and Smith 2001), and, most importantly, to provide extensive data regarding the methods that are sufficient and necessary to produce error rates and statistical confidences excellent enough for practical application in the field.

In the original papers on the specific set of scientific methods that has come to be called “brain fingerprinting,” Farwell and Donchin (1991)^2^ and Farwell and Smith (2001) reported a 0 % error rate and high statistical confidence for all determinations. To make our statistical statements more conservative, and for the purpose of meaningful mathematical comparisons, in our discussions herein, we will generally exaggerate the error rate slightly and use “less than 1 %” to characterize the error rate in studies where in fact a 0 % error rate was obtained. In addition to 0 % error rate, Farwell and Smith also reported 0 % indeterminates. Replications of these methods in other laboratories, e.g., Allen and Iacono (1997), have achieved similar results (see also Iacono 2007, 2008; Iacono and Lykken 1997; Iacono and Patrick 2006; Neshige et al. 1991). These are the methods that were ruled admissible in court in the Harrington case (Erickson 2007; Farwell and Makeig 2005; Harrington v. State 2001; Roberts 2007; Farwell 2013). For a comprehensive tutorial review of all related publications in English to date, see Farwell (2012).

In the criminal justice system and in national security, error rate is of paramount importance. In the United States, error rate is specified as one of the fundamental tests of the prevailing Daubert standard for admissibility in court (Farwell and Makeig 2005; Roberts 2007). Accuracy is 100 % minus the error rate. For a discussion of these terms and their legal and scientific implications, see Farwell (2012).

Some subsequent studies have been based on fundamentally different scientific principles, have applied fundamentally different methods, and consequently have produced substantially different results. They have reported error rates approximately 10–50 times higher than those of the original brain fingerprinting studies, as well as susceptibility to countermeasures. For a comprehensive review, see Farwell (2012). For example, Rosenfeld et al. (2004) reported an overall 35 % error rate in detecting information-present subjects (65 % accuracy) without countermeasures and 67 % error rate (33 % accuracy) with countermeasures for their various techniques and conditions. Error rates of their various methods in detecting information-present subjects ranged from 8 to 46 % without countermeasures, and from 61 to 82 % with countermeasures. In a series of studies, the “complex trial protocol” (Rosenfeld et al. 2008) has produced an overall error rate of 15 % without countermeasures and 29 % with countermeasures. For a review, see Farwell (2012). (See also Farwell 2011a, b).

Farwell (2012) described in detail the brain fingerprinting methods that have produced less than 1 % error rate and high statistical confidence, and discussed the reasons that various alternative methods that have produced error rates an order of magnitude or more higher and statistical confidences substantially lower. That paper also discussed the specific methodological shortcomings that led to susceptibility to countermeasures in various alternative techniques that did not follow the methods applied in our original research and the present research.

In our view, in order to be viable for field use or any other application with major consequences, a technique must produce an overall error rate of less than 1 % in all studies and field applications, an error rate of less than 5 % in every individual study, and a record of consistently high statistical confidences for both information-present and information-absent determinations—averaging at least 90 % for information-present determinations and 90 % in the opposite direction for information-absent determinations, and preferably averaging over 95 % in the correct direction for all determinations of both types. To make a decision in a specific field case with judicial or other life-changing consequences, in our view the statistical confidence for the determination should be at least 95 %, whether it is information present or information absent. In our actual field applications, every individual determination to date has been with at least 99 % statistical confidence with the P300-MERMER.

The present four studies were designed to test the hypothesis that following the standard scientific procedures specified in Farwell and Donchin (1991), Farwell and Smith (2001), and Farwell (1992, 1994, 1995a, b, 2007, 2010, 2012) is sufficient to produce valid and reliable results, consistently less than 1 % error rates, and extremely high statistical confidence for each determination. We tested this hypothesis in demanding field conditions involving real-life crimes and life-changing consequences of the outcome of the tests, including judicial consequences such as the death penalty or life in prison, and in other cases a $100,000 reward for beating the test. In one study we also tested the hypothesis that these specific brain fingerprinting methods are unaffected by the countermeasures that have proven effective against alternative techniques (e.g., Rosenfeld et al. 2004, 2008; Mertens and Allen 2008).

In these studies we compared the performance of two data analysis methods involving different but overlapping time epochs and the corresponding event-related brain responses. One included only the P300, which has been known for over half a century as consisting of a positive peak. The other included both the P300 and a later negative peak (late negative potential or LNP) that follows the P300 in the data collected in our laboratory and in other laboratories applying the same paradigm. The P300 and the LNP together we refer to as a P300-MERMER (memory and encoding related multifaceted electroencephalographic response). The characteristics of this response are described in Farwell (1994, 1995b, 2007, 2010), in Farwell and Smith (2001), and in more detail in Farwell (2012).

### Four brain fingerprinting field/real-life studies

The present report comprises four brain fingerprinting field/real-life studies. In all four studies we used brain fingerprinting to detect information obtained in the course of real-life events. Studies 1 and 2 used specific issue brain fingerprinting tests to detect specific issue information regarding real-life events, including capital crimes. Studies 3 and 4 used specific screening brain fingerprinting tests to detect real-life specific group knowledge of FBI agents and experts in bomb making, i.e., explosive ordnance disposal (EOD) and improvised explosive devices (IEDs).

In Study 1, the “CIA Real Life Study,” brain fingerprinting was used to detect concealed information regarding real-life events, including a number of felony crimes. There were, however, no significant consequences of the outcome of the tests, and consequently no substantial motivations for subjects.

In Study 2, the “Real Crime Real Consequences $100,000 Reward Study,” brain fingerprinting was used to detect information regarding real crimes. In some cases, the subjects were highly motivated because they were facing either the death penalty or life imprisonment, and the brain fingerprinting test detected presence or absence of information regarding the crime in question. In cases where there was less inherent motivation resulting from a potential judicial outcome, subjects were offered a $100,000 reward for beating the test. Except in cases where life and freedom were at stake, subjects were taught countermeasures that have previously proved effective against other, fundamentally different, non-brain fingerprinting techniques (Mertens and Allen 2008; Rosenfeld et al. 2004) but not against brain fingerprinting (see Farwell 2011a, b, 2012).

In Study 3, the “FBI Agent Study,” brain fingerprinting was used to detect information that is known to FBI agents but not to the general public, such as FBI techniques, procedures, acronyms, information learned in FBI training, etc.

In Study 4, the “Bomb Maker Study,” brain fingerprinting was used to detect information that is known to explosive ordnance disposal (EOD) and improvised explosive device (IED) experts but not to the general public.

In a fifth study, we set out to apply an alternative, non-brain fingerprinting “complex trial protocol” in detecting real-life information with some of the same subjects as Study 2. We discontinued this study for scientific and ethical reasons, as explained in the Discussion section. (For details, see Farwell 2012.)

### P300 and P300-MERMER

The original brain fingerprinting research (Farwell 1992; Farwell and Donchin 1986, 1991) used the P300 component of the event-related brain potential (ERP). The P300 is a positive voltage potential maximal at the midline parietal scalp (Pz in the International 10–20 System) that peaks at 300 or more milliseconds from the onset of the eliciting event (Donchin et al. 1986; Farwell and Donchin 1988a; Miller et al. 1987; Sutton et al. 1965). Farwell and colleagues (Farwell 1994, 1995b, 2012; Farwell and Smith 2001) have shown that in the brain fingerprinting paradigm this positive peak is followed by a late negative peak (the Late Negative Potential or LNP). The two together have been termed P300-MERMER (memory and encoding related multifaceted electroencephalographic response). Both the P300 and the P300-MERMER undoubtedly have other features beyond the simple time-domain pattern that becomes apparent through the usual ERP signal-averaging procedures (Farwell 1994; Farwell and Smith 2001; see also Rapp et al., 1993). The positive–negative-peaked pattern in the time domain (or negative–positive–negative pattern if the N2 preceding the P300 is included), however, is sufficient to define the response, and is all that is necessary to detect it. This time-domain analysis is all that is used in our data analysis in the present paper. Data analysis in the present paper compares the results obtained by including only the P300 in the analysis with the results obtained by including the full P300-MERMER in the analysis. In both cases, only the usual time-domain characteristics of the signals that are used in conventional ERP analysis are considered in the computations. The only difference is the length of the epoch analyzed.

When we first discovered the P300-MERMER, specifically the late negative peak (LNP) that follows the positive P300 peak in the full P300-MERMER, our initial hypothesis was that the LNP of the MERMER was an artifact, possibly caused by the effect of the analog filters used in data collection on the return of the P300 to baseline. We soon discovered that the artifact hypothesis is not supported by the data. Experimentation (including recording without analog filters), scalp distribution (the relative amplitude at different scalp sites), and morphology (the latency and shape of the waveforms) has proven that the LNP of the P300-MERMER is not an artifact of the signal-detection or noise-reduction procedures or equipment, such as digital and analog filters or of the return of the P300 to baseline (Farwell 1994, 1995b, 2012).

To definitively test the hypothesis that the LNP of the P300-MERMER was a filter-generated artifact, we recorded without analog filters. We found that recording without analog filters did not diminish the amplitude of the LNP or change its latency, thus disproving the filter-generated artifact hypothesis.

Moreover, the data recorded with filters are also incompatible with the hypothesis that the LNP of the P300-MERMER is an artifact of any kind. The recording equipment is identical for all scalp sites and all subjects. If the LNP were an artifact of the equipment, the identical equipment would produce the same effects in different instances. The characteristics of the LNP would simply be a function of the P300. On the contrary, we found that the relative latency, amplitude, and morphology of the P300 and the LNP are very different for different subjects and for different scalp sites in the same subject. In different subjects, we found that virtually identical P300s were followed by LNPs that differed in latency by hundreds of milliseconds and differed in amplitude by a factor of two or more. In a number of cases the LNP was substantially smaller than the P300 at one channel (usually Pz) and substantially larger than the P300 at another channel (usually Fz) for the same subject in the same data set. These data are incompatible with the hypothesis that the LNP of the P300-MERMER is simply an artifact generated by some combination of the P300, the return to baseline after the P300, and the recording equipment and filters.

The positive P300 peak (or two peaks—P3a and P3b) is preceded by a negative peak, the N200, and followed by another negative peak, the LNP, producing a tri-phasic shape for the P300-MERMER. We first observed this triphasic negative–positive–negative pattern at the scalp in the early 1990s (Farwell 1994, 1995b, 2012; Farwell and Smith 2001). The same negative–positive–negative pattern has been observed in intracranial recordings in various structures (Halgren et al. 1998), including the inferior parietal lobe/supramarginal gyrus, superior temporal sulcus (Halgren et al. 1995), the amygdala and hippocampus (Halgren et al. 1986; Stapleton and Halgren 1987), dorsolateral and orbital frontal cortices, and the anterior cingulate (Baudena et al. 1995).

In short, the P300-MERMER is not simply the P300 followed by an artifact. It is a response produced in the brain that includes a negative peak following the positive P300 peak. By now virtually all of the researchers involved in detection of concealed information with brainwaves include in their computational algorithms both the positive P300 and the late negative potential (LNP) that constitutes the other major facet of the P300-MERMER (for a review, see Farwell 2012). Differences in nomenclature still exist, however. Some use the term the “amplitude of the P300” to refer to what we call the amplitude of the P300-MERMER, that is, the sum of the amplitudes of the P300 and the LNP. Computationally this is the voltage difference between the most positive point in the P300 time range and the most negative point in the LNP time range. In any case, we use all points in the entire waveform in our computations, not just the peaks, so the question of nomenclature is moot. Our data analysis matches patterns, and it does not matter what the responses are called.

### The term “Brain Fingerprinting”

The term “brain fingerprinting” is based on the defining feature of matching something on the person of the suspect with something from the crime scene. Fingerprinting matches prints at the crime scene with prints on the fingers of the suspect. DNA “fingerprinting” matches biological samples from the crime scene with biological samples from the suspect. “Brain fingerprinting” matches information stored in the brain of the suspect with information from the crime scene. We use the term “brain fingerprinting” to refer to any methods, beginning with the original Farwell and Donchin (1991), Farwell (1992), and Farwell and Smith (2001) studies, that meet or exceed all or almost all the brain fingerprinting scientific standards specified below. Brain fingerprinting studies analyze the data based on either the P300-MERMER, or the P300 alone, or both (as in the present study).

## Methods

### Standard methods for all four studies

Previous publications (Farwell 1992, 1994, 1995a, b, 2007, 2010, 2012; Farwell and Donchin 1991; Farwell and Smith 2001) specify the standard methods we have applied in all our brain fingerprinting studies. For a detailed account of the scientific principles and specific methods, see Farwell (2012). These methods are briefly summarized below.

Three types of stimuli consisting of words or phrases are presented on a computer screen. (Pictures and auditory stimuli may also be used, but were not in these studies.) Probe stimuli contain specific information relevant to the investigated situation. The test is designed to detect the subject’s knowledge or lack of knowledge of the probes as relevant in the context of the crime or other investigated situation. (We shall generally refer to the investigated situation as a “crime,” although of course other, non-criminal situations can be investigated, as in Studies 3 and 4.) Probes have the following defining characteristics.

1. Probes contain features of the crime or investigated situation that in the judgment of the criminal investigator the perpetrators would have experienced in committing the crime, or the subjects would have learned in the course of gaining the specific knowledge, training, or expertise investigated;
2. Probes contain information that the subject has no way of knowing if he did not participate in the crime or other situation of interest; and
3. Probes contain information that the subject claims not to know or to recognize as significant for any reason.

In order to test whether or not the subject recognizes the probes as significant in the context of the investigated situation, we present two additional types of stimuli. Target stimuli elicit a response that provides a standard for the subject’s brain response to known information relevant to the investigated situation. Irrelevants elicit a response that provides a standard for the subject’s response to irrelevant, unknown information.

Target stimuli present situation-relevant information that is known to be known to the subject. This information may have been revealed to the subject through news reports, interrogation, etc. In any case, the targets are disclosed to the subject before the test. The subject instructions also convey the significance of each target in the context of the investigated situation.

Irrelevant stimuli contain plausible, but incorrect, information about the crime. For a subject lacking the relevant knowledge contained in the probes, the irrelevants and probes are equally plausible as crime-relevant details. For each probe (and each target) several irrelevants are structured that contain similar but incorrect information. For example, if a probe is the murder weapon, a pistol, corresponding irrelevants could be a rifle, a shotgun, and a knife. The subject is informed of the significance of the probes in the context of the investigated situation (e.g., “the murder weapon”), but is not informed which is the correct, crime-relevant probe and which are the corresponding irrelevants.

The previous research that has produced a 0 % error rate and extremely high statistical confidence for each determination was conducted according to the following scientific standards for brain fingerprinting tests. The present studies also met these standards.

### Scientific standards for brain fingerprinting tests

The following procedures comprise the scientific standards for brain fingerprinting tests (Farwell 1992, 1994, 1995a, b, 2007, 2010, 2012; Farwell and Donchin 1991; Farwell and Smith 2001; Harrington v. State 2001).

1. Use equipment and methods for stimulus presentation, data acquisition, and data recording that are within the standards for the field of cognitive psychophysiology and event-related brain potential research. These standards are well documented elsewhere. For example, the standard procedures Farwell introduced as evidence in the Harrington case were accepted by the court, the scientific journals, and the other expert witnesses in the case. Use a recording epoch long enough to include the full P300-MERMER. For pictorial stimuli or realistic word stimuli, use at least a 1,800-ms recording epoch. (Shorter epochs may be appropriate for very simple stimuli.)
2. Use correct electrode placement. The P300 and P300-MERMER are universally known to be maximal at the midline parietal scalp site, Pz in the standard International 10–20 System.
3. Apply brain fingerprinting tests only when there is sufficient information that is known only to the perpetrator and investigators. Use a minimum of six probes and six targets.
4. Use stimuli that isolate the critical variable: the subject’s knowledge or lack of knowledge of the probe stimuli as significant in the context of the investigated situation. Obtain the relevant knowledge from the criminal investigator (or for laboratory studies from the knowledge-imparting procedure such as a mock crime and/or subject training session). Divide the relevant knowledge into probe stimuli and target stimuli. Probe stimuli constitute information that has not been revealed to the subject. Target stimuli contain information that has been revealed to the subject after the crime or investigated situation.
5. If initially there are fewer targets than probes, create more targets. Ideally, this is done by seeking additional known information from the criminal investigators. Note that targets may contain information that has been publicly disclosed. Alternatively, some potential probe stimuli can be used as targets by disclosing to the subject the specific items and their significance in the context of the investigated situation.
6. For each probe and each target, fabricate several stimuli of the same type that are unrelated to the investigated situation. These become the irrelevant stimuli. Use stimuli that isolate the critical variable. For irrelevant stimuli, select items that would be equally plausible for a non-knowledgeable subject. The stimulus ratio is approximately one-sixth probes, one-sixth targets, and two-thirds irrelevants.
7. Ascertain that the probes contain information that the subject has no known way of knowing, other than participation in the investigated situation. This information is provided by the criminal investigator for field studies, and results from proper information control in laboratory studies.
8. Make certain that the subject understands the significance of the probes, and ascertain that the probes constitute only information that the subject denies knowing, as follows. Describe the significance of each probe to the subject. Show him the probe and the corresponding irrelevants, without revealing which is the probe. Ask the subject if he knows (for any non-crime-related reason) which stimulus in each group is crime-relevant. Describe the significance of the probes and targets that will appear in each test block immediately before the block.
9. If a subject has knowledge of any probes for a reason unrelated to the investigated situation, eliminate these from the stimulus set. This provides the subject with an opportunity to disclose any knowledge of the probes that he may have for any innocent reason previously unknown to the scientist. This will prevent any non-incriminating knowledge from being included in the test.
10. Ascertain that the subject knows the targets and their significance in the context of the investigated situation. Show him a list of the targets. Describe the significance of each target to the subject.
11. Require an overt behavioral task that requires the subject to recognize and process every stimulus, specifically including the probe stimuli, and to prove behaviorally that he has done so on every trial. Detect the resulting brain responses. Do not depend on detecting brain responses to assigned tasks that the subject can covertly avoid doing while performing the necessary overt responses.
12. Instruct the subjects to press one button in response to targets, and another button in response to all other stimuli. Do not instruct the subjects to “lie” or “tell the truth” in response to stimuli. Do not assign different behavioral responses or mental tasks for probe and irrelevant stimuli.
13. In order to obtain statistically robust results for each individual case, present a sufficient number of trials of each type to obtain adequate signal-to-noise enhancement through signal averaging. Use robust signal-processing and noise-reduction techniques, including appropriate digital filters and artifact-detection algorithms. The number of trials required will vary depending on the complexity of the stimuli, and is generally more for a field case. In their seminal study, Farwell and Donchin (1991) used 144 probe trials. In the Harrington field case, Farwell used 288 probe trials (Harrington v. State 2001). In any case, use at least 100 probe trials and an equal number of targets. Present three to six unique probes in each block.
14. Use appropriate mathematical and statistical procedures to analyze the data. Do not classify the responses according to subjective judgments. Use statistical procedures properly and reasonably. At a minimum, do not determine subjects to be in a category where the statistics applied show that the determination is more likely than not to be incorrect.
15. Use a mathematical classification algorithm, such as bootstrapping on correlations, that isolates the critical variable by classifying the responses to the probe stimuli as being either more similar to the target responses or to the irrelevant responses. In a forensic setting, conduct two analyses: one using only the P300 (to be more certain of meeting the standard of general acceptance in the scientific community), and one using the P300-MERMER (to provide the current state of the art).
16. Use a mathematical data-analysis algorithm that takes into account the variability across single trials, such as bootstrapping.
17. Set a specific, reasonable statistical criterion for an information-present determination and a separate, specific, reasonable statistical criterion for an information-absent determination. Classify results that do not meet either criterion as indeterminate. Recognize that indeterminate outcome is not an error, neither a false positive nor a false negative.
18. Restrict scientific conclusions to a determination as to whether or not a subject has the specific situation-relevant knowledge embodied in the probes stored in his brain. Recognize that brain fingerprinting detects only presence or absence of information—not guilt, honesty, lying, or any action or non-action. Do not offer scientific opinions on whether the subject is lying or whether he committed a crime or other act. Recognize that the question of guilt or innocence is a legal determination to be made by a judge and jury, not a scientific determination to be made by a scientist or a computer.
19. Evaluate error rate/accuracy based on actual ground truth. Ground truth is the true state of what a scientific test seeks to detect. Brain fingerprinting is a method to detect information stored in a subject’s brain. Ground truth is whether the specific information tested is in fact stored in the subject’s brain. Establish ground truth with certainty through post-test interviews in laboratory experiments and in field experiments wherein subjects are cooperative. Establish ground truth insofar as possible through secondary means in real-life forensic applications with uncooperative subjects. Recognize that ground truth is the true state of what the subject in fact knows, not what the experimenter thinks the subject should know, not what the subject has done or not done, and not whether the subject is guilty, or deceptive.
20. Make scientific determinations based on brain responses. Do not attempt to make scientific determinations based on overt behavior that can be manipulated, such as reaction time.

### Error rate/accuracy standards for field applications

In the United States and many other jurisdictions, the error rate of a scientific technique is critical for admissibility as scientific evidence in court. The error rate is the percentage of determinations made that are either false negatives or false positives. In brain fingerprinting, this is the percentage of “information present” and “information absent” determinations that are false positives and false negatives respectively.

In our view, in order to be viable for field use or any other application with major consequences, a technique must produce an overall error rate of less than 1 % in all studies and field applications, an error rate of less than 5 % in every individual study, and a record of consistently high statistical confidences for both information-present and information-absent determinations—averaging at least 90 % for information-present determinations and 90 % in the opposite direction for information-absent determinations, and preferably averaging over 95 % in the correct direction for all determinations of both types. To make a decision in a specific field case with judicial or other life-changing consequences, in our view the statistical confidence for the determination should be at least 95 %, whether it is information present or information absent. In our actual field applications, every individual determination to date has been with at least 99 % statistical confidence with the P300-MERMER.

Alternative methods that do not meet the above scientific standards have generally produced error rates at least ten times higher than this standard (e.g., Rosenfeld et al. 2004, 2008). Some methods that fail to meet the standards have consistently produced statistical confidences no better than chance for information-absent determinations (e.g., Rosenfeld et al. 2004, 2008).

All four of the experiments reported here followed the above overall methods. All four experiments met the brain fingerprinting scientific standards 1–20 described above.

All subjects signed informed consent forms. All procedures were approved by Brain Fingerprinting Laboratories, Inc.’s Institutional Review Board. Specific methods for each of the four studies are described below.

### Study 1: the CIA Real Life Study

The CIA Real Life Study was a specific issue test. The information detected consisted of specifics regarding particular events in the lives of the subjects. In some but not all cases these life experiences included felony crimes. All of the tests, however, were conducted in circumstances where there were no judicial consequences of the outcome of the tests. Subjects were assured of confidentiality.

In the “information present” cases, probes were words or phrases associated with an event in the subject’s life. In three of the 20 cases the subjects were “information absent,” i.e., none of the stimuli were relevant to the subject. Their probe stimuli were the probe stimuli that were relevant for another subject.

Target stimuli were also relevant to the investigated event. Target stimuli, unlike probes, were identified to the subject in the course of experimental instructions. The target items were made relevant to all subjects by naming each target stimulus, explaining its relevance to the crime or investigated situation, and instructing the subject to press a special button only in response to targets. Subjects were instructed to press a button with one thumb in response to targets, and another button with the other thumb in response to all other stimuli. The prediction was that targets would elicit a P300-MERMER in all subjects, irrelevants would not elicit a P300-MERMER, and probes would elicit a P300-MERMER only in information-present subjects.

Information for structuring the stimuli was obtained from interviews with someone familiar with each subject. Subjects knew the identity of the informant for their case, and had given permission for the information to be provided for the purpose of research. As is the case in actual criminal investigations, subjects did not discuss the events and information to be detected with the experimenter prior to the testing session, or give any indication of having participated in the events or of knowing the relevant information. However, unlike the situation in actual criminal investigations, subjects were assured that results would be kept confidential. CIA Real Life Study results were not used in any legal proceedings. (In our second field study, as described immediately below, however, brain fingerprinting results were used in criminal cases and as evidence admitted in judicial proceedings in court.)

The probe stimuli were not identified as probes to the subjects. Subjects gave no behavioral indication of knowing the information contained in the probes. All stimuli were presented on a computer monitor under computer control, according to prearranged parameters that were identical for all subjects and for all stimuli. Data analysis was conducted using a standard signal-processing and mathematical analysis procedure for all subjects.

In the “information absent” cases, none of the probe items were relevant to the subject. All of the probe items were relevant to one of the other subjects. The target items were made relevant to the subject as described above. Subjects were instructed to press one button for targets and another button for all other stimuli. These “other” stimuli constituted irrelevant and probe stimuli. Unlike the information-present subjects, however, the information-absent subjects did not recognize the probes. For them, probes were indistinguishable from irrelevants, since the subjects lacked the relevant knowledge contained in the probes.

Stimuli were constructed in groups of six: one probe, one target, and four irrelevants. For each probe stimulus there were two similar irrelevant stimuli, and for each target stimulus there were two similar irrelevant stimuli. The stimuli were structured such that each probe and its similar irrelevants were indistinguishable for a subject lacking the information that the test was structured to reveal. That is, if a given probe was an article of clothing relevant to the crime or situation under investigation, two articles of clothing irrelevant to the crime were presented; if a particular probe stimulus was a name, there were two irrelevant stimuli that were also names, and so on. Similarly, there were two irrelevant stimuli that corresponded to each target.

For each subject, there were nine unique probes, nine unique targets, and 36 unique irrelevants, a total of 54 unique stimuli. These comprised nine groups of stimuli, each consisting of one probe, one target, and four irrelevants.

Testing was divided into separate blocks. In each block the computer display presented 72 stimulus presentations or trials. Three stimulus groups were presented in each block, that is, in each block there were three unique probes, three unique targets, and 12 unique irrelevants. Each stimulus was presented four times in a block to make the total of 72 stimulus presentations per block. Stimuli were presented in random order.

Stimuli were presented for a duration of 300 ms at an inter-stimulus interval of 3,000 ms. A fixation point was presented for 1,000 ms prior to each stimulus presentation.

Trials contaminated by artifacts generated by eye movements or other muscle-generated noise were rejected on-line, and additional trials were presented so that the required number of 72 artifact-free trials was obtained. The criterion for artifact rejection was as follows: trials with a range of greater than 97.7 μV in the EOG channel were rejected. This is discussed and illustrated in more detail in Appendix 3.

After three blocks, all nine groups of stimuli had been presented once. Blocks 4–6 then presented the same stimuli as blocks 1–3 respectively. The stimuli were repeated again in blocks 6–9. Thus, each of the 54 unique stimuli appeared in three different blocks, for a total of 12 presentations of each unique stimulus, and a grand total of 648 trials. Of these, 108 were probe trials, 108 were target trials, and 432 were irrelevant trials. Subjects had a rest period of approximately 2 min between blocks.

Stimulus presentation, data acquisition, and data analysis were accomplished with a PC-based system using custom software. One monitor presented the stimuli to the subject. A second monitor presented a display to the operator. During data acquisition, the display included continuous data from four channels in real time; continually updated averages of the three trial types overplotted; artifact data including values at each channel for threshold, range, slope, and mean absolute deviation, with a change in color when a rejection criterion was exceeded; reaction time and accuracy for each trial and averaged by trial type; information on the stimulus presented for each trial; counts of total and artifact-free trials by trial type; and additional information.

Brain responses were recorded from the midline frontal, central, and parietal scalp locations (Fz, Cz, and Pz respectively, International 10–20 System) referenced to linked mastoids (behind the ear), and from a location on the forehead to track eye movements. (Eye movements generate scalp potentials that interfere with the brain potentials being recorded.) Med Associates silver–silver chloride disposable electrodes were held in place by a custom headband.

Data were digitized at 333 Hz, and resampled at 100 Hz off-line for analysis. Electroencephalograph (EEG) data were amplified at a gain of 50,000 using custom amplifiers. Electro-oculograph (EOG/eye movement) data were amplified at a gain of 10,000. Impedance did not exceed 10 kilohm. Analog filters passed signals between .1 and 30 Hz. Data were stored on disk for off-line data analysis.

The primary data analysis task in these experiments was to determine whether the responses to the probe stimuli, like the responses to the target stimuli, contained a P300-MERMER brainwave pattern. We used bootstrapping (Farwell and Donchin 1988b; Wasserman and Bockenholt 1989) to determine whether the probe responses were more similar to the target responses or to the irrelevant responses, and to compute a statistical confidence for this determination for each individual subject. The bootstrapping procedure is described in more detail in Appendix 1. Appendix 2 provides graphic illustrations of key steps in the bootstrapping procedure.

We used bootstrapping to estimate the sampling distribution of two correlations: the correlation between the average of the probe trials and the average of the irrelevant trials, and the correlation between the probe average and the target average. In our computations we used “double-centered” correlations (i.e., the grand mean for all trials of all types was subtracted from the probe, target, and irrelevant average waveforms prior to the correlation computations). If the correlation between the probe and target trials is significantly greater than the correlation between the probe and irrelevant trials, then we can conclude that the probe brain responses are more similar to the targets (where a P300-MERMER is present) than to the irrelevants (where there is no P300-MERMER). If this is the case, then we can conclude that the subject recognizes the probes as a separate, rare category—that is, of situation-relevant events—and therefore that the subject is knowledgeable regarding the investigated situation. Similarly, if the correlation between the probe and irrelevant trials is greater than the correlation between the probe and target trials, then we can conclude that the subject lacks this information.

For each subject we computed the percentage of iterations in which the probe-target correlation was greater than the probe-irrelevant correlation. This provided the bootstrap index, or statistical confidence for an information present determination. This is the statistical probability that the probes, like the targets, contain the P300-MERMER or P300 pattern of interest. The bootstrap index for an information-absent determination is the percentage of iterations where the probe-irrelevant correlation is greater than the probe-target correlation. This is the probability that an information-absent determination is correct. This is equivalent to 100 % minus the probability for the information-present determination. That is, an information-present confidence of 99 % (that is, 99 % probability that the information present determination is correct) is equivalent to an information-absent confidence of 1 % (that is, 1 % probability that an information-absent determination is correct).

A decision regarding the status (information present or information absent) of a given subject depends on comparing his/her bootstrap index with criterion levels for information-present and information-absent determinations. The a priori criteria for information-present and information-absent determinations were set at 90 and 70 % respectively. These criteria were arrived at on the basis of the results of previous research (Farwell 1992; Farwell and Donchin 1991; Farwell and Smith 2001).

Prior to analysis, data were digitally filtered using a 49-point, equal-ripple, zero-phase-shift, optimal, finite impulse response, low-pass filter with a passband cutoff frequency of 6 Hz and a stopband cutoff frequency of 8 Hz (Farwell et al. 1993). Trials with eye-movement or muscle-generated artifacts were rejected by a signal-detection algorithm prior to analysis. Trials with a range of greater than 97.7 μV in the EOG channel were rejected.

We conducted two separate analyses on each subject. One analysis used the P300-MERMER, consisting of the positive P300 peak followed by the late negative peak (LNP). A second analysis included only the positive P300 peak. The P300-MERMER epoch was defined as 300–1,800 ms after the onset of the stimulus. The P300 epoch was defined as 300–900 ms after the onset of the stimulus. For subjects with markedly shorter or longer latencies than the norm, a more precise definition was applied using the target response as a template, as follows. The P300 epoch was defined as the epoch between 300 and 900 ms where the target response was more positive than the irrelevant response. The P300-MERMER epoch was defined as the P300 epoch followed by the epoch where the target response was more negative (or less positive) than the irrelevant response.

The data analysis algorithm produced two sets of results for each subject: a determination of information present or information absent and a statistical confidence for the determination using the full P300-MERMER, and a similar determination and statistical confidence using the P300 alone. This allowed us to compare the error rate/accuracy and statistical confidence provided by the state-of-the-art P300-MERMER as compared with the more widely known and well established P300.

Brain fingerprinting is a test to detect information stored in the brain. The accuracy of any system for detecting concealed information (or anything else) can only be meaningfully evaluated in light of ground truth. Ground truth is by definition the true state of exactly what the procedure is attempting to discover. For any detection method in any science, ground truth is the factual, real-world truth regarding whether the item to be detected is actually present at the time that the detection method is applied. If one conducts a DNA test to determine whether sample A matches sample B, ground truth is whether the two samples actually do in fact represent the same DNA. (Ground truth is not, for example, whether or not the suspect is guilty of a crime.)

For brain fingerprinting, ground truth is whether or not the relevant information is stored in the subject’s brain at the time of the test. Specifically, ground truth is whether or not the subject knows the information contained in the probe stimuli at the time of the test. Ground truth is not whether the subject is guilty of a crime, whether the subject participated in a knowledge-imparting procedure such as a mock crime, or whether the experimenter (or anyone else) thinks the subject should, could, or would know the information contained in the probe stimuli if he did or did not commit a crime or for any other reason. In particular, ground truth is not what the experimenter knows, or what the experimenter thinks the subject should know, or what the subject has done or not done. Ground truth is the true state of the subject–knowledgeable (information present) regarding the information contained in the probes, or not—at the time of the test.

Ground truth was established by post-test interviews. All subjects were cooperative and were not facing adverse consequences from the outcome of the test. Therefore it was possible to establish ground truth with a high degree of certainty through post-test interviews. The significance of each probe stimulus was described to each subject in post-test interviews, and the subject was asked to identify the correct probe stimulus. Post-test interviews established that all information-present subjects knew the information contained in all the probe stimuli, and no information-absent subjects knew the information contained in any of the probe stimuli. The correctness of determinations was evaluated in light of ground truth.

### Study 2: the Real Crime Real Consequences $100,000 Reward Study

The Real Crime Real Consequences $100,000 Reward Study was a specific issue study involving real-world events with real, substantial consequences. We tested brain fingerprinting on 14 subjects^3^ in circumstances where subjects were highly motivated by real-world consequences of the outcome of the tests. We used brain fingerprinting to detect concealed information regarding real crimes, in circumstances where the outcome of the test could produce major, life-changing consequences. Some of the subjects were suspects in criminal investigations or convicted prisoners who claimed innocence and were appealing their convictions. In some cases the subjects were facing the death penalty or life in prison, and the outcome of the brain fingerprinting test could provide legally admissible evidence relevant to the case and the ensuing consequences.

In some cases, although the crimes were real, there were no reasonably foreseeable, life-changing legal consequences of the outcome of the brain fingerprinting test. To produce a life-changing impact in cases where no judicial outcome hinged on the scientific results, we offered subjects a $100,000 reward for beating the test. Beating the test means producing a false negative result: producing an information-absent determination when the subject knew the relevant knowledge, so the correct determination would have been information present.

Such subjects were taught countermeasures that had previously proven effective against other, fundamentally different, non-brain fingerprinting tests (but not against brain fingerprinting; see Farwell 2011a, b, 2012). One countermeasure (Rosenfeld et al. 2004) involves instructing subjects to attempt to enhance responses to irrelevant stimuli. This is done by dividing the irrelevant stimuli into categories and performing a specific covert act such as wiggling the big toe in the left shoe in response to certain specific categories. An alternative countermeasure involves instructing subjects to attempt to enhance responses to target stimuli by thinking of being slapped or applying pressure to their toes in response to each target stimulus (Mertens and Allen 2008). We taught subjects one or the other of these countermeasures, using identical subject instructions to those applied in Rosenfeld et al. and Mertens and Allen.

Probe stimuli were obtained by the criminal investigators involved through the usual evidence-collection procedures involved in criminal investigations, including interviewing witnesses and accomplices, inspecting the crime scene, examining police reports and other investigative reports, reviewing court records, etc. Target stimuli were obtained through similar sources, and also through publicly available information such as news reports.

Unlike the procedures in the CIA Real Life Study, confidentiality in the Real Crime Real Consequences $100,000 Reward Study was not maintained in cases where the brain fingerprinting results were relevant to a current criminal case. Scientific reports and expert testimony were provided as appropriate in relevant judicial proceedings. Subjects were informed of this in advance, and all subjects signed informed consent forms. For the purpose of this report, however, individual subject confidentiality is maintained.

The purpose of the brain fingerprinting test in each unsolved criminal case was to determine whether or not the information contained in the probe stimuli, provided by the criminal investigator as putative features of the crime, was stored in the brain of the subject. The brain fingerprinting determinations and statistical confidence, and the resulting reports and expert witness testimony in court, addressed only the question of whether the specific crime-relevant information contained in the probes was known to the subject.

Brain fingerprinting testing, and the brain fingerprinting scientists, did not provide an opinion regarding the guilt or innocence of the subject. The brain fingerprinting test did not address, and the brain fingerprinting scientists did not opine regarding, the effectiveness of the criminal investigation, the relevance of the probes to the crime, or the probative value of the brain fingerprinting results with respect to the question of who committed the crime.

Attorneys and prosecutors on both sides did debate these matters, referring to common sense, life experience, and other sources outside the realm of science to support their contentions. These are non-scientific issues that are decided by a judge and jury based on their human judgment, life experience, and common sense. The question of guilt or innocence is decided by a judge and jury, not by a scientist or a computer. The brain fingerprinting test results simply provided the judge and jury with additional evidence that they weighed along with the other evidence in reaching their findings of fact regarding what took place at the time of the crime and their legal verdicts regarding guilt or innocence.

Data acquisition and data analysis methods were the same as described for the CIA Real Life Experiment, except for the following. The number of probes, the number of data acquisition blocks, and the timing of the tests varied depending on the individual circumstances of the various cases. To provide ample data even in adverse circumstances, all subjects were scheduled to be tested in 25 blocks of 72 trials each. In some cases tests were conducted in prisons, and modifications were necessary to meet prison scheduling requirements and other logistical issues. Some tests actually consisted of fewer blocks and fewer trials than the number originally scheduled, but in no case fewer than the methods for the CIA Real Life Study described above. The reference was linked ears. Digitizing rate was 100 Hz.

Brain fingerprinting is a test to detect information stored in the brain. As discussed above, ground truth is the true state of whatever the test is attempting to determine. With brain fingerprinting, ground truth is whether or not the subject knew the information contained in the probe stimuli at the time of the test. In field studies involving any forensic science, it is never possible to establish ground truth with absolute certainty. Our study is no exception. Neither we nor any other forensic scientist applying any forensic science test in the real world can know with absolute certainty what ground truth is.

For information-present subjects, ground truth was that the crime-relevant information contained in the probe stimuli was stored in the brain of the subject at the time of the test. Culpability for the crimes was established with a relatively high degree of certainty through confessions, corroborated in every case with judicial outcome when relevant. Confessions, particularly when combined with convictions, can establish a reasonably high degree of confidence (although not an absolute certainty) that the subject is guilty of the crime. This does not establish ground truth, however, because ground truth for brain fingerprinting is not whether the subject is guilty but whether the subject knows the information contained in the probes.

The only way to establish ground truth with absolute certainty is for the subject to correctly identify the probes in post-test interviews, without ever having been told which stimuli are the correct, crime-relevant probes. Fortunately, all our information-present subjects eventually confessed and cooperated. In post-test interviews, we described the significance of each of the probe stimuli in the context of the crime, and asked the subject to identify the correct probe stimulus. All of the information-present subjects correctly identified all of the probe stimuli. Obviously, a subject can do this only if ground truth is that he does know the crime-relevant information contained in each probe. Thus, ground truth was established with certainty for all of our information-present subjects. The correctness of determinations was evaluated in the light of real-world ground truth.

When ground truth is the absence of something, this can never be absolutely proven without some kind of assumption or circular reasoning. Just because people have looked almost everywhere and no one has ever found a pink elephant does not absolutely prove that no such thing exists.

In the case of information-absent subjects, ground truth was that the information contained in the probes was not stored in their brains at the time of the test. For our subjects, in every case someone else confessed and/or was convicted of the crime for which the probe stimuli were relevant, and sworn witness testimony, compelling physical evidence, and judicial findings of fact held that our subject was not present at the crime scene. This established with a rather high degree of certainty that the subject did not know the relevant information through participation in the crime.

The remaining possibility was that the subject somehow knew the information contained in the probes for an innocent reason. This information had never been publicly released, and court and investigative records stated that the subject had never been exposed to the information. Moreover, subjects were provided an opportunity to disclose any knowledge of the information contained in the probes that they may have found out though some unknown innocent means. The significance of each probe stimulus was clearly described to the subjects, and they were asked if they knew the correct crime-relevant information for any reason. They had an extremely high motivation to disclose any such innocently acquired knowledge if it existed, because such disclosure would provide an innocent reason why they would be found to possess crime-relevant information that would imminently be detected by the brain fingerprinting test. No subjects offered any innocent reason (or any reason) why they might know any of the information contained in any of the probes.

The probability is vanishingly small that a subject knew specific never-released information about a crime for which someone else had confessed and been convicted, when official records and judicial findings of fact established that he had no possible known way of knowing the information, and when he had opportunity and extremely high motivation to reveal such knowledge if it had been acquired through some previously unknown innocent means. Although, as in all field studies in any forensic science, ground truth cannot be established with absolute certainty, ground truth was established with a high degree of certainty for all information-absent subjects. The correctness of determinations was evaluated in the light of real-world ground truth.

### Study 3: the FBI Agent Study

The FBI Agent Study was a specific screening study. The relevant information detected was information known to FBI agents but not to the general public, obtained from interviews with FBI agents. We tested 17 FBI agents (information present) and four non-agents (information absent).

Data acquisition methods were the same as for the above studies, except for the following. Stimuli were visually presented words, phrases, and acronyms that are well known to FBI agents and not to the general public. There were a total of 33 probes, 33 targets, and 132 irrelevants. Testing consisted of six blocks of 72 trials each. In each block subjects viewed either five or six sets of stimuli, each set consisting of one probe, one target, and four irrelevants.

The reference was linked mastoids. Digitizing rate was 100 Hz.

Data analysis methods were the same as in the above described studies, except for the difference in recording and analysis epoch described below.

Ground truth was whether the subject knew the FBI-relevant information contained in the probes at the time of the test. Since all subjects were fully cooperative, ground truth could be established by post-test interviews. Post-test interviews established that all information-present subjects knew all of the probes. No information-absent subjects knew any of the probes.

The FBI Agent Study was the first time brain fingerprinting was used to detect specific group knowledge rather than specific issue knowledge. This study also resulted in two other innovations, as follows.

The FBI Agent study was the first study in which we used targets that contained information relevant to the investigated situation (in this case, inside knowledge relevant to the FBI). In previous studies we had used targets that were irrelevant to the investigated situation, and were made relevant only by subject instructions that informed the subjects which stimuli were targets and required them to push one button in response to targets and another button in response to all other stimuli.

Using target stimuli which, like the probes, are known and recognized as significant for an information-present subject increases the accuracy of the system. Recall that the data analysis involves comparing the probe-target correlation with the probe-irrelevant correlation. For an information-present subject, any procedure that maximizes the probe-target correlation and/or minimizes the probe-irrelevant correlation will improve discrimination and increase accuracy.

Situation-relevant targets improve the accuracy of the system particularly when the stimuli are acronyms, as some of them were in both the FBI Agent Study and the Bomb Maker Study. In these studies, only acronyms were presented in some blocks, and only words and phrases were presented in other blocks.

Consider the case of an information-present subject when the probe stimuli are acronyms known to the subject and the targets and irrelevants are both meaningless letter strings. Both targets and probes are relevant and noteworthy, so both will elicit large P300-MERMERs. However, the information-present subjects will be able to identify the probes more quickly than the targets and irrelevants. They can immediately recognize probes as acronyms. Targets and irrelevants, by contrast, are both meaningless letter strings. When the stimulus is not an immediately recognizable probe, subjects must search through the strings to determine if they are among the target strings they have been given. Since they can recognize the probes more quickly, the latency of the P300-MERMER to the probes will tend to be less than the latency of the P300-MERMER to both targets and irrelevants. The result is that the targets will resemble the probes in P300-MERMER amplitude (i.e., large) but will resemble the irrelevants in P300-MERMER latency (i.e., long). When correlations are computed between the responses to the respective trial types, the correlation between the targets and the probes—which should be high in an information present subject because both elicit large P300-MERMERs—will tend to be attenuated because of the latency difference. The peaks will not line up. The result is that the determinations rendered by the system will tend to be less accurate.

When targets are relevant acronyms known to an information present subject, the P300-MERMER latency differences serve to improve discrimination. In this situation, an information-present subject can quickly recognize both targets and probes as known acronyms. Therefore both targets and probes will elicit P300-MERMERs with short latency (as well as high amplitude). The irrelevants, being meaningless letter strings, will be deciphered more slowly, and will elicit a longer latency response (with a very small P300-MERMER, if any). As before, both probes and targets will elicit relatively high amplitude P300-MERMERs. Thus, the correlation between targets and probes will be high because both have large P300-MERMERs of similar (short) latency. The correlation between probes and irrelevants will tend to be lower not only because the irrelevants have a small P300-MERMER (if any) but also because the P300-MERMER latencies do not match—irrelevant brain-response latencies are longer than probes. A higher correlation between targets and probes, combined with a lower correlation between probes and irrelevants, will increase the probability of a correct information-present determination.

This same phenomenon takes place, albeit to a lesser degree, when the stimuli are words or phrases. Using crime-relevant words or phrases maximizes the similarity between the probe and target stimuli, and hence the similarity between the probe and target brain responses, for an information-present subject. This maximizes the probe-target correlation if, and only if, the subject is factually information present, which increases accuracy and statistical confidence. Standard 4 includes this feature.

All of the above applies only to an information-present subject. For a subject without the relevant information, using targets that are relevant acronyms will be no different than using meaningless strings, because she will not recognize the acronyms. For an information-absent subject, all of the stimuli will be perceived as meaningless letter strings. Thus, no latency differences between trial types are to be expected. The discrimination will be made on the basis of amplitude. For such a subject, the targets are noteworthy solely because she has been instructed to press a special button only for targets. The target P300-MERMER amplitude will be greater than that for probes and irrelevants, as usual for an information-absent subject. Thus the probes will resemble the irrelevants rather than the targets.

The second innovation of the FBI Agent Study was that it was the first study in which we recorded a long enough data epoch to observe the full late negative potential (LNP) of the P300-MERMER. In early studies, P300 research had used relatively short stimuli such as clicks, tones, and single words or short phrases. P300 researchers typically presented a stimulus every 1–1.5 s. In our previous brain fingerprinting work, for example, we (Farwell 1992; Farwell and Donchin 1991) used an inter-stimulus interval of 1,500 ms.

In the FBI Agent Study we were required to use stimuli that accurately represented knowledge of the FBI. It was not practical to use only short words and short phrases. Some of the stimuli consisted of several words of several syllables each. To give the subjects time to recognize and process the stimuli, we extended the inter-stimulus interval to 3,000 ms. As is described in the Discussion section, we discovered that when the stimuli are longer phrases, which take some time for the subject to decipher, and the inter-stimulus interval is sufficiently long to display the full response, the positive P300 peak is followed by a late negative peak (LNP). The LNP has a peak latency sometimes as long as 1,500 ms, and it sometimes does not resolve to baseline until around 1,800 ms. We called this full response, including both the P300 and the LNP, a memory and encoding related multifaceted electroencephalographic response or P300-MERMER. Other features of the P300-MERMER are discussed in the Discussion section and in Farwell (1994, 1995b, 2007, 2010, 2012) and Farwell and Smith (2001).

Because when we designed the study we had not yet discovered this late negative peak (LNP) of the P300-MERMER, we recorded and analyzed a shorter data epoch than in the above described studies (which we conducted subsequently). In the FBI agent study, the data recording and analysis epoch ended at 1,250 ms after stimulus onset. In subsequent studies we have used a 1,800-ms data analysis epoch. (We also record continuous data.)

### Study 4: the Bomb Maker Study

The Bomb Maker Study was a specific screening study. The information detected was information that is known to individuals with extensive experience in making, detecting, detonating, and deactivating or destroying bombs. Information present subjects were explosive ordnance disposal (EOD) and improvised explosive device (IED) experts. We obtained the relevant information that comprised the probe and target stimuli through interviews with EOD/IED experts. We tested 17 information-present subjects and two information-absent subjects. Stimuli were words, phrases, and acronyms that are known to EOD/IED experts and not to the general public.

Data acquisition methods were the same as for the above studies, except for the following.

For each subject, there were 21 unique probes, 21 unique targets, and 84 unique irrelevants, a total of 126 unique stimuli. These comprised 21 groups of stimuli, each consisting of one probe, one target, and four irrelevants.

Testing was divided into separate blocks. In each block, either four or five stimulus groups were presented, that is, in each block there were either four unique probes, four unique targets, and 16 unique irrelevants, or five probes, five targets, and 20 irrelevants. Each block consisted of 72 trials, or individual stimulus presentations, with presentations of unique stimuli repeated as necessary to reach this total. We presented 10 blocks for each subject. Thus, we presented a total of 720 trials for each subject, including 120 probe trials, 120 target trials, and 480 irrelevant trials.

In block 1 we presented the first five stimulus groups, i.e., five unique probes, five unique targets, and 20 unique irrelevants. In each of blocks 2–5 we presented four of the remaining stimulus groups. At the end of the first five blocks, each of the 21 stimulus groups had been presented in one block. The same pattern was repeated for blocks 6–10.

The reference was linked ears. Digitizing rate was 100 Hz.

Data analysis methods were the same as in the above described studies.

Ground truth was whether the subject knew the bomb-making relevant information contained in the probes at the time of the test. Since all subjects were fully cooperative, ground truth could be established by post-test interviews. Post-test interviews established that all information-present subjects knew all of the probes. No information-absent subjects knew any of the probes.

## Results

### Overview of results for the four brain fingerprinting studies

The target stimuli elicited a large P300-MERMER in all subjects. This is as expected, since the targets contained known, relevant information for all subjects. Also as expected, the irrelevant stimuli did not elicit a large P300-MERMER in any subjects. As predicted, the probe stimuli elicited a large P300-MERMER only in the information-present subjects, and not in the information-absent subjects. In the information-present subjects, the response to the crime-relevant (or situation-relevant) probes was similar to the response to the known targets: both contained a large P300-MERMER. In the information-absent subjects, the response to the crime-relevant (or situation-relevant) probes was similar to the response to the irrelevant stimuli: neither contained a large P300-MERMER.

The brain fingerprinting data-analysis algorithm using the P300-MERMER produced the following overall results: Error rate was 0 %. 100 % of determinations were correct. There were no false negatives and no false positives. There were also no indeterminates. As in Farwell and Donchin (1991), Farwell and Smith (2001), and all other previous brain fingerprinting research, Grier (1971) A’ was 1.0.

The criterion for an information-present determination was a 90 % statistical confidence, computed by bootstrapping. The criterion for an information-absent determination was a 70 % statistical confidence in the opposite direction. All determinations with the P300-MERMER, both information-present and information-absent, achieved over 95 % statistical confidence. In studies 1 and 2, the specific issue studies, all determinations with the P300-MERMER achieved at least 99 % confidence. The median statistical confidence for the individual determinations with the P300-MERMER in all studies was 99.9 %. The mean statistical confidence for the individual determinations with the P300-MERMER in all studies was 99.5 %.

The brain fingerprinting data-analysis algorithm using the P300 alone produced the same determinations: 100 % of determinations were correct. Error rate was 0 %. Accuracy was 100 %.^4^ There were no false negatives and no false positives. Also, there were no indeterminates. The median statistical confidence for the individual determinations with the P300 in all studies was 99.6 %. The mean statistical confidence for the individual determinations with the P300 in all studies was 97.9 %. All determinations with the P300, both information-present and information-absent, achieved over 90 % statistical confidence.

The P300-MERMER produced significantly higher statistical confidences than the P300 alone, *p* < .0001 (sign test). For most of the subjects (57 %), the statistical confidence for the P300-MERMER-based determination was higher than the statistical confidence for the P300-based determination.

Table 1 summarizes the error rate/accuracy of brain fingerprinting determinations for all subjects in all four studies combined.

**Table 1:** Brain fingerprinting accuracy/error rate all studies

Correct positives	62	100 %
Correct negatives	14	100 %
Total correct determinations	76	100 %
False positives	0	0 %
False negatives	0	0 %
Indeterminates	0	0 %
Accuracy	76/76	100 %
Error rate	0/76	0 %

Table 2 summarizes the brain fingerprinting determinations for all subjects in all four studies.

**Table 2:** Brain fingerprinting determinations—all studies

Determination	Subject state		
	Information present	Information absent	Total
Information present	62	0	62
Information absent	0	14	14
Indeterminate	0	0	0
Total	62	14	76

### Study 1: the CIA Real Life Study

Table 3 summarizes the error rate and accuracy of brain fingerprinting determinations in the CIA Real Life Study.

**Table 3:** Brain fingerprinting accuracy/error rate CIA Real Life Study

Correct positives	17	100 %
Correct negatives	3	100 %
Total correct determinations	20	100 %
False positives	0	0 %
False negatives	0	0 %
Indeterminates	0	0 %
Accuracy	20/20	100 %
Error rate	0/20	0 %

Table 4 summarizes the brain fingerprinting determinations for the CIA Real Life Study.

**Table 4:** Brain fingerprinting determinations—CIA Real Life Study

Determination	Subject state		
	Information present	Information absent	Total
Information present	17	0	17
Information absent	0	3	3
Indeterminate	0	0	0
Total	17	3	20

Table 5 presents the determinations and statistical confidence for information-present subjects in the CIA Real Life Study.

**Table 5:** Brain Fingerprinting determinations and statistical confidence—CIA Real Life Study information-present subjects

Subject #	P300-MERMER analysis		P300 analysis		Improvement of P300-MERMER over P300 (%)
	Determination	Statistical confidence (%)	Determination	Statistical confidence (%)	
1	IP	99.9	IP	99.1	0.8
2	IP	99.9	IP	99.9	0.0
3	IP	99.9	IP	99.9	0.0
4	IP	99.9	IP	99.2	0.7
5	IP	99.9	IP	99.9	0.0
6	IP	99.9	IP	99.9	0.0
7	IP	98.6	IP	93.6	5.0
8	IP	99.9	IP	99.4	0.5
9	IP	99.9	IP	99.9	0.0
10	IP	99.9	IP	99.9	0.0
11	IP	99.9	IP	99.9	0.0
12	IP	99.9	IP	99.9	0.0
13	IP	99.9	IP	99.7	0.2
14	IP	99.9	IP	99.8	0.1
15	IP	99.9	IP	95.8	4.1
16	IP	99.9	IP	99.9	0.0
17	IP	99.9	IP	99.9	0.0

*IP* information present. *IA* information absent

Table 6 presents the determinations and statistical confidence for information-absent subjects in the CIA Real Life Study.

**Table 6:** Brain fingerprinting determinations and statistical confidence CIA Real Life Study—information-absent subjects

Subject #	P300-MERMER analysis		P300 analysis		Improvement of P300-MERMER over P300 (%)
	Determination	Statistical confidence (%)	Determination	Statistical confidence (%)	
18	IA	99.9	IA	99.6	0.3
19	IA	99.9	IA	99.6	0.3
20	IA	99.9	IA	99.8	0.1

*IP* information present. *IA* information absent

**Fig. 1:**
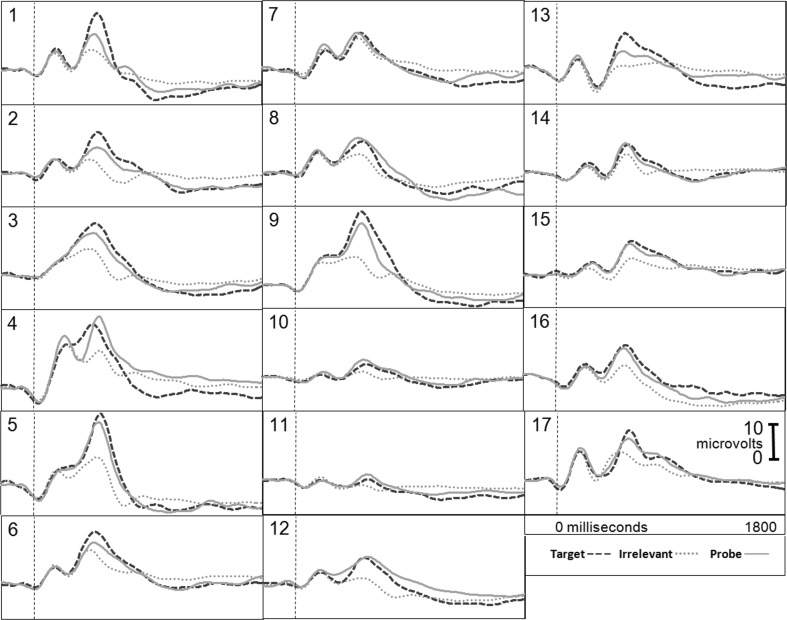
CIA Real Life Study brain responses: information-present subjects

Figure 1 presents the brain responses for information-present subjects in the CIA Real Life Study.

**Fig. 2:**
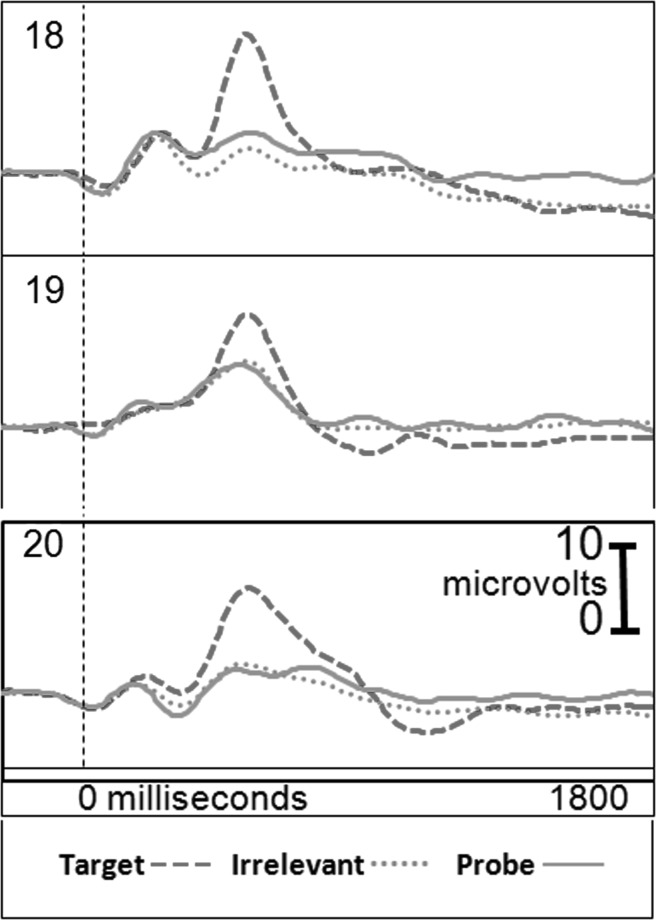
CIA Real Life Study brain responses: information-absent subjects

Figure 2 presents the brain responses for information-absent subjects in the CIA Real Life Study.

Tables 3, 4, 5 and 6 present the following overall results for the CIA Real Life Study. Error rate was 0 %. Accuracy was 100 %: 100 % of determinations were correct. There were no false negatives and no false positives. There were also no indeterminates. All determinations were made with a very high statistical confidence (Tables 5, 6). In this study, all but one of the determinations with the P300-MERMER had a statistical confidence of 99.9 %. (One determination was at a 98.6 % confidence, still well above the 90 % criterion).

Not only were all determinations correct; none of the determinations were close to indeterminate. All of the determinations were very far statistically from either a false positive or a false negative. All determinations were grouped in the 0.1 or 1.4 % range at the correct end of the theoretical distribution of bootstrap values. The least statistically confident information-present determination and the least statistically confident information-absent determination were separated by a buffer of over 98 percentage points in the bootstrap statistic.

### Study 2: the Real Crime Real Consequences $100,000 Reward Study

Table 7 summarizes the error rate/accuracy of brain fingerprinting determinations in the Real Crime Real Consequences $100,000 Reward Study.

**Table 7:** Brain fingerprinting accuracy/error rate Real Crime Real Consequences $100,000 Reward Study

Correct positives	9	100 %
Correct negatives	5	100 %
Total correct determinations	14	100 %
False positives	0	0 %
False negatives	0	0 %
Indeterminates	0	0 %
Accuracy	14/14	100 %
Error rate	0/14	0 %

Table 8 summarizes the determinations for the Real Crime Real Consequences $100,000 Reward Study.

**Table 8:** Brain fingerprinting determinations Real Crime Real Consequences $100,000 Reward Study

Determination	Subject state		
	Information present	Information absent	Total
Information present	9	0	9
Information absent	0	5	5
Indeterminate	0	0	0
Total	9	5	14

Table 9 presents the determinations and statistical confidence for information-present subjects in the Real Crime Real Consequences $100,000 Reward Study.

**Table 9:** Brain fingerprinting determinations and statistical confidence Real Crime Real Consequences $100,000 Reward Study—information-present subjects

Subject #	P300-MERMER analysis		P300 analysis		Improvement of P300-MERMER over P300 (%)
	Determination	Statistical confidence (%)	Determination	Statistical confidence (%)	
1	IP	99.9	IP	99.9	0.0
2	IP	99.9	IP	99.7	0.2
3	IP	99.9	IP	99.9	0.0
4	IP	99.9	IP	99.4	0.5
5	IP	99.0	IP	97.5	1.5
6	IP	99.9	IP	99.9	0.0
7	IP	99.9	IP	99.2	0.7
8	IP	99.9	IP	99.9	0.0
9	IP	99.9	IP	99.7	0.3

*IP* information present. *IA* information absent

Table 10 presents the determinations and statistical confidence for information-absent subjects in the Real Crime Real Consequences $100,000 Reward Study.

**Table 10:** Brain fingerprinting determinations and statistical confidence Real Crime Real Consequences $100,000 Reward Study—information-absent subjects

Subject #	P300-MERMER analysis		P300 analysis		Improvement of P300-MERMER over P300 (%)
	Determination	Statistical confidence (%)	Determination	Statistical confidence (%)	
10	IA	99.9	IA	99.5	0.4
11	IA	99.9	IA	91.5	8.4
12	IA	99.5	IA	99.5	0.0
13	IA	99.9	IA	99.7	0.2
14	IA	99.9	IA	99.9	0.0

*IP* information present. *IA* information absent

**Fig. 3:**
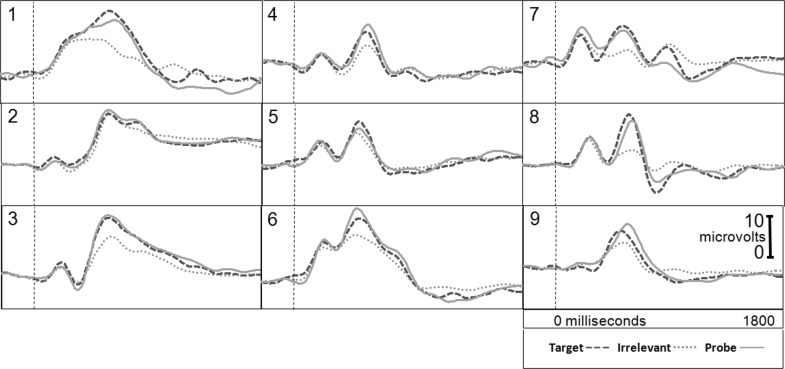
Real Crime Real Consequences $100,000 Reward Study brain responses: information-present subjects

Figure 3 presents the brain responses for information-present subjects in the Real Crime Real Consequences $100,000 Reward Study.

**Fig. 4:**
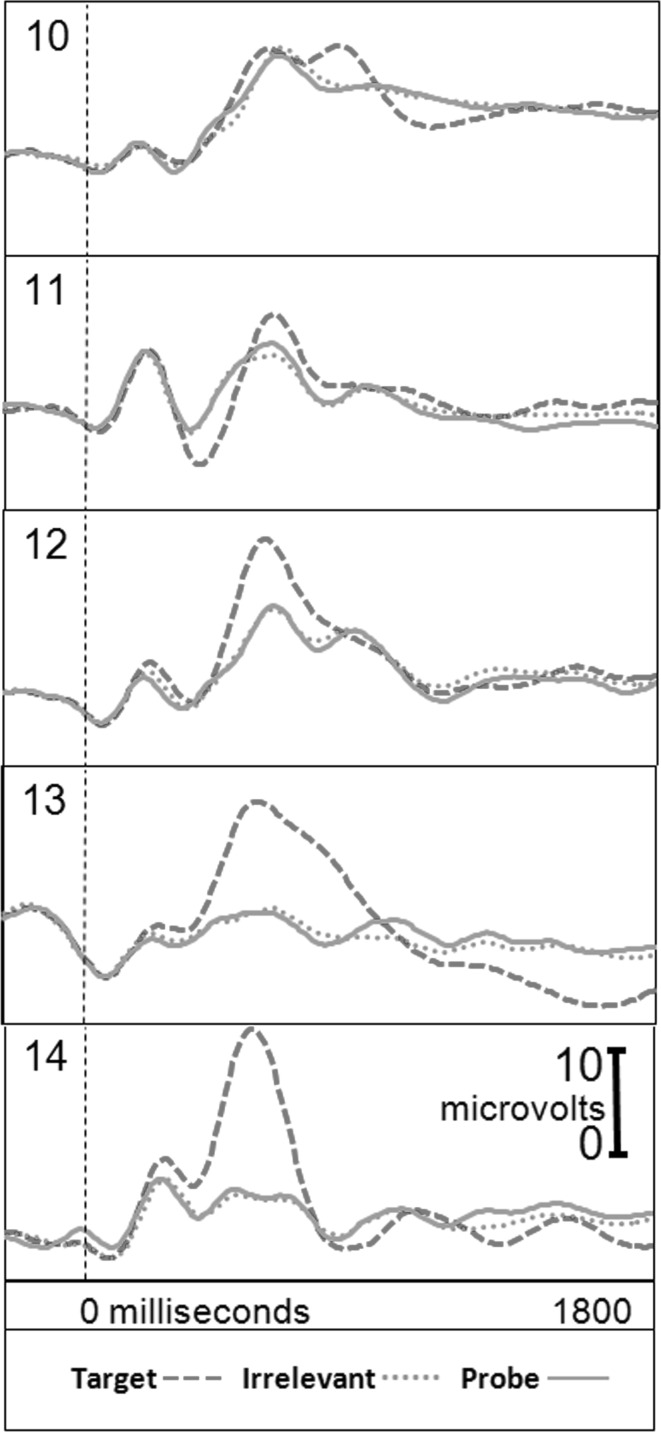
Real Crime Real Consequences $100,000 Reward Study brain responses: information-absent subjects

Figure 4 presents the brain responses for information-absent subjects in the Real Crime Real Consequences $100,000 Reward Study.

Tables 7, 8, 9 and 10 present the following overall results for the Real Crime Real Consequences $100,000 Reward Study. Determinations were highly accurate, and were achieved with a high level of statistical confidence. Error rate was 0 %. Accuracy was 100 %: 100 % of determinations were correct. There were no false positives and no false negatives. There were also no indeterminates. The median statistical confidence for determinations with the P300-MERMER was 99.9 %. The mean statistical confidence was 99.8 %. All but two of the determinations were made with a statistical confidence of 99.9 %. All determinations had a statistical confidence of at least 99 % (Tables 9, 10), well above the criteria for information-present or information-absent determinations (90 and 70 % respectively).

No determinations were close to indeterminate, and all determinations were very far statistically from a false negative or a false positive. Not only were 100 % of determinations correct; all determinations were grouped in the correct 1 % range at one end or the other of the theoretically possible distribution of the bootstrap statistic. The least statistically confident information-present determination and the least statistically confident information-absent determination were separated by a buffer of 98 percentage points in the bootstrap statistic.

### Study 3: the FBI Agent Study

Table 11 summarizes the error rate/accuracy of brain fingerprinting determinations in the FBI Agent Study.

**Table 11:** Brain fingerprinting accuracy/error rate FBI Agent Study

Correct positives	17	100 %
Correct negatives	4	100 %
Total correct determinations	21	100 %
False positives	0	0 %
False negatives	0	0 %
Indeterminates	0	0 %
Accuracy	21/21	100 %
Error rate	0/21	0 %

Table 12 summarizes the brain fingerprinting determinations for the FBI Agent Study.

**Table 12:** Brain fingerprinting determinations—FBI Agent Study

Determination	Subject state		
	Information present	Information absent	Total
Information present	17	0	17
Information absent	0	4	4
Indeterminate	0	0	0
Total	17	4	21

Table 13 presents the determinations and statistical confidence for information-present subjects in the FBI Agent Study.

**Table 13:** Brain fingerprinting determinations and statistical confidence FBI Agent Study—information-present subjects

Subject #	P300-MERMER analysis		P300 analysis		Improvement of P300-MERMER over P300 (%)
	Determination	Statistical confidence (%)	Determination	Statistical confidence (%)	
1	IP	99.9	IP	99.9	0.0
2	IP	99.9	IP	99.9	0.0
3	IP	99.9	IP	94.9	5.0
4	IP	99.9	IP	99.9	0.0
5	IP	99.9	IP	99.9	0.0
6	IP	99.6	IP	99.6	0.0
7	IP	99.9	IP	99.9	0.0
8	IP	99.2	IP	98.4	0.5
9	IP	99.9	IP	99.9	1.2
10	IP	99.6	IP	94.6	1.0
11	IP	99.0	IP	90.7	7.3
12	IP	99.8	IP	99.8	0.0
13	IP	99.9	IP	99.9	9.4
14	IP	99.9	IP	98.3	1.0
15	IP	99.9	IP	99.9	8.7
16	IP	99.9	IP	98.1	1.7
17	IP	99.9	IP	92.7	2.3

*IP* information present. *IA* information absent

Table 14 presents the determinations and statistical confidence for information-absent subjects in the FBI Agent Study.

**Table 14:** Brain fingerprinting determinations and statistical confidence FBI Agent Study—information-absent subjects

Subject #	P300-MERMER analysis		P300 analysis		Improvement of P300-MERMER over P300 (%)
	Determination	Statistical confidence (%)	Determination	Statistical confidence (%)	
18	IA	98.2	IA	93.8	4.4
19	IA	97.1	IA	91.8	5.3
20	IA	99.0	IA	95.0	4.0
21	IA	96.4	IA	93.5	2.9

*IP* information present. *IA* information absent

**Fig. 5:**
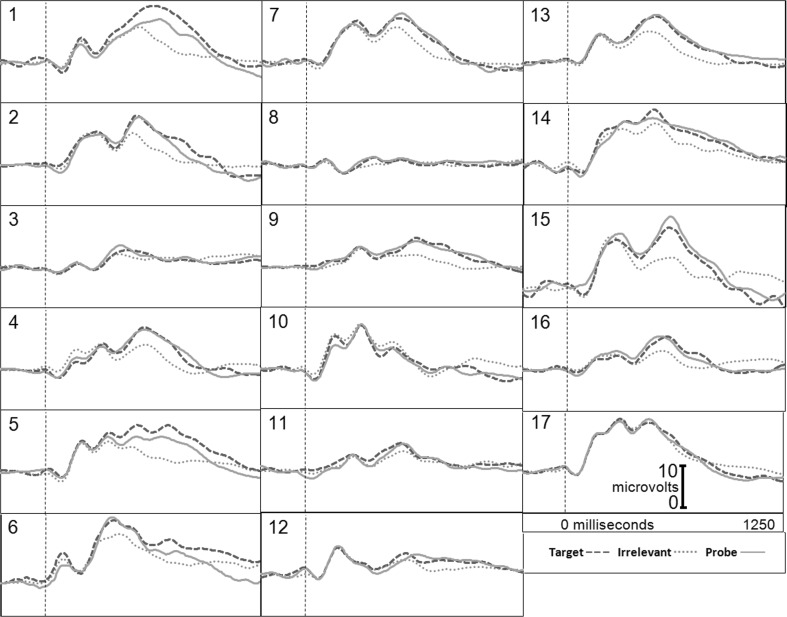
FBI Agent Study brain responses: information-present subjects

Figure 5 presents the brain responses for information-present subjects in the FBI Agent Study.

**Fig. 6:**
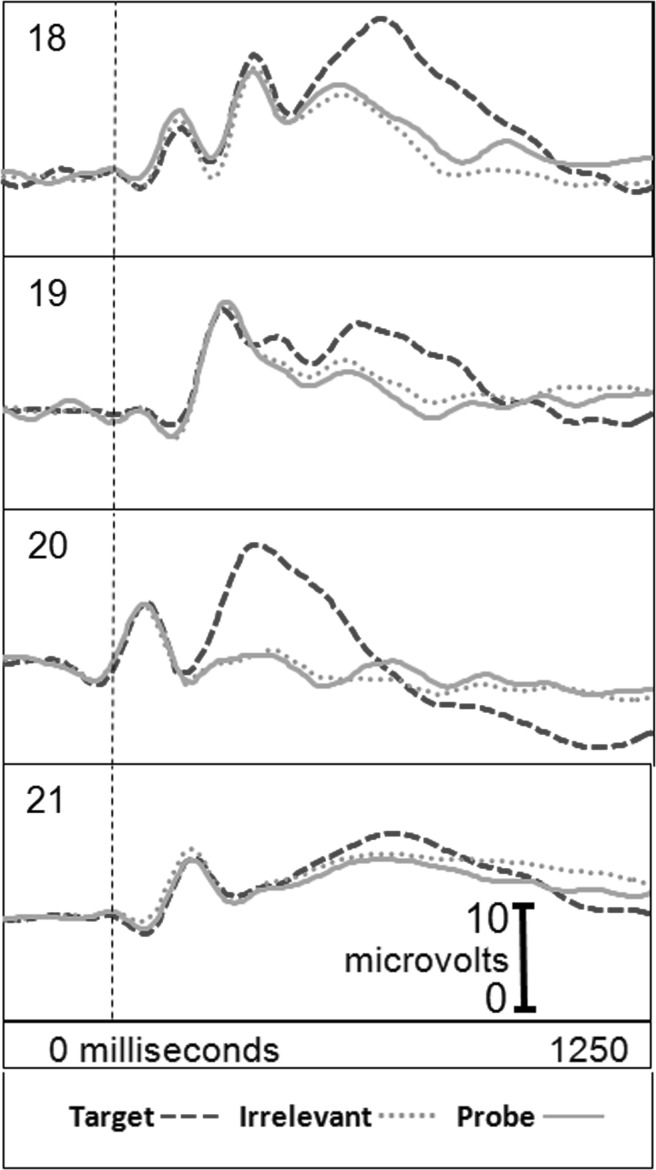
FBI Agent Study brain responses: information-absent subjects

Figure 6 presents the brain responses for information-absent subjects in the FBI Agent Study.

Tables 11, 12, 13 and 14 present the following overall results for the FBI Agent Study. Error rate was 0 %; accuracy was 100 %: 100 % of determinations were correct. There were no false negatives and no false positives, and also no indeterminates. Statistical confidence was high in every case. The median statistical confidence was 99.9 % for the P300-MERMER analysis. That is, most of the statistical confidences for determinations with the P300-MERMER were 99.9 %, and all information-present determinations were over 99 % (Table 13). Information-absent determinations were also 100 % accurate. Statistical confidence for information-absent determinations was not quite as high, but still over 96 % in every case, well above the criterion (Table 14).

Not only were determinations 100 % accurate, none of the determinations were close statistically to being indeterminate, and all of the determinations were very far statistically from a false negative or a false positive. All determinations were grouped at the correct 4 % or 1 %, at the correct end of the theoretical distribution of the bootstrap statistic. The least confident information-present determination and the least confident information-absent determination were separated by a buffer of over 95 percentage points in the bootstrap statistic.

### Study 4: the Bomb Maker Study

Table 15 summarizes the error rate and accuracy of determinations in the Bomb Maker Study.

**Table 15:** Brain fingerprinting accuracy/error rate Bomb Maker Study

Correct positives	19	100 %
Correct negatives	2	100 %
Total correct determinations	21	100 %
False positives	0	0 %
False negatives	0	0 %
Indeterminates	0	0 %
Accuracy	21/21	100 %
Error rate	0/21	0 %

Table 16 summarizes the determinations for the Bomb Maker Study.

**Table 16:** Brain fingerprinting determinations—Bomb Maker Study

Determination	Subject state		
	Information present	Information absent	Total
Information present	19	0	19
Information absent	0	2	2
Indeterminate	0	0	0
Total	19	2	21

Table 17 presents the determinations and statistical confidence for information-present subjects in the Bomb Maker Study.

**Table 17:** Brain fingerprinting determinations and statistical confidence Bomb Maker Study—information-present subjects

Subject #	P300-MERMER Analysis		P300 Analysis		Improvement of P300-MERMER over P300 (%)
	Determination	Statistical confidence (%)	Determination	Statistical confidence (%)	
1	IP	99.9	IP	99.9	0.0
2	IP	99.9	IP	99.9	0.0
3	IP	99.9	IP	99.5	0.4
4	IP	99.9	IP	99.9	0.0
5	IP	97.7	IP	93.4	4.3
6	IP	99.7	IP	94.1	5.6
7	IP	99.9	IP	99.9	0.0
8	IP	99.9	IP	99.4	0.5
9	IP	95.4	IP	94.2	1.2
10	IP	95.2	IP	94.2	1.0
11	IP	99.5	IP	92.2	7.3
12	IP	99.9	IP	99.9	0.0
13	IP	99.9	IP	90.5	9.4
14	IP	99.9	IP	98.9	1.0
15	IP	99.9	IP	91.2	8.7
16	IP	99.9	IP	98.2	1.7
17	IP	99.9	IP	97.6	2.3
18	IP	99.9	IP	98.9	1.0
19	IP	99.9	IP	98.3	1.6

*IP* information present. *IA* information absent

Table 18 presents the determinations and statistical confidence for information-absent subjects in the Bomb Maker Study.

**Table 18:** Brain fingerprinting determinations and statistical confidence Bomb Maker Study—information-absent subjects

Subject #	P300-MERMER analysis		P300 analysis		Improvement of P300-MERMER over P300 (%)
	Determination	Statistical confidence (%)	Determination	Statistical confidence (%)	
20	IA	97.2	IA	97.2	0.0
21	IA	95.0	IA	95.0	0.0

*IP* information present. *IA* information absent

**Fig. 7:**
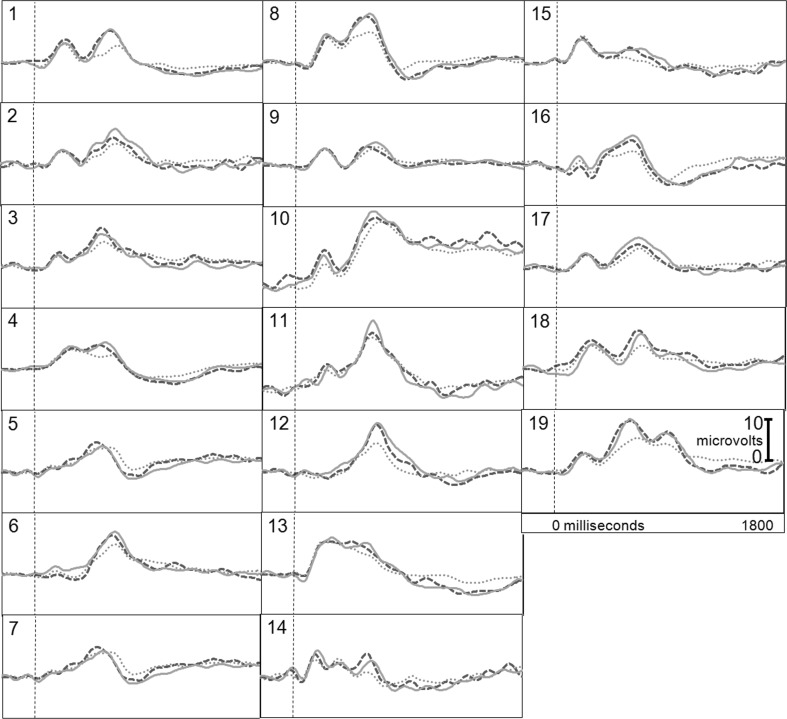
Bomb Maker Study brain responses: information-present subjects

Figure 7 presents the brain responses for information-present subjects in the Bomb Maker Study.

**Fig. 8:**
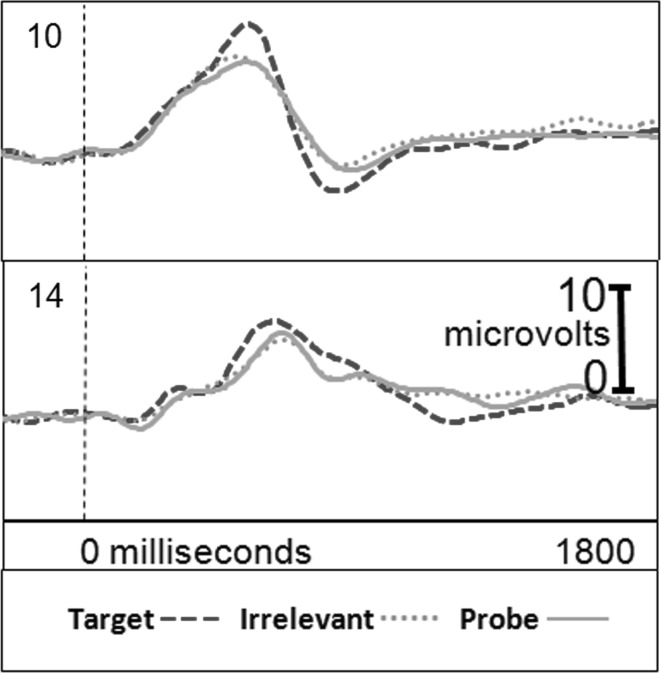
Bomb Maker Study brain responses: information-absent subjects

Figure 8 presents the brain responses for information-absent subjects in the Bomb Maker Study.

Tables 15, 16, 17 and 18 present the following overall results for the Bomb Maker Study. Error rate was 0 %. Accuracy was 100 %: 100 % of determinations were correct. There were no false negatives and no false positives, and also no indeterminates. The median statistical confidence for P300-MERMER-based determinations was 99.9 %. 14 of the 21 determinations (67 %) were made with a statistical confidence of 99.9 %.

Information-present determinations were generally achieved with a higher statistical confidence than information-absent determinations. All P300-MERMER-based determinations were made with at least a 95 % confidence (Tables 17, 18), well above the criteria.

As with the other studies, no determination was anywhere near a false negative or a false positive. All determinations were grouped in the correct 5 % at each end of the theoretical distribution of the bootstrap statistic. The least confident information-present determination and the least confident information-absent determination were separated by a buffer of over 90 percentage points. This provides a very high level of confidence that all of the brain fingerprinting results are far from either a false negative or a false positive.

### Summary of results of the P300-MERMER-based analysis

In all four brain fingerprinting studies, error rate was 0 %. Accuracy was 100 %. 100 % of determinations were correct. There were no false negatives and no false positives. There were also no indeterminates. We used a priori criteria of 90 % statistical confidence for an information-present determination and 70 % statistical confidence for an information-absent determination. For the analysis based on the P300-MERMER, all of the determinations were in fact considerably above these pre-established criteria. All information-present determinations were over 95 % confidence, five percentage points above the criterion in the bootstrap statistic. All information-absent determinations were also over 95 % confidence, 25 percentage points above the criterion.

All determinations were much further from a false negative or a false positive than from an indeterminate result. Recall that an information-present determination with a statistical confidence or probability of 95 % is equivalent to a 5 % probability (that is, 100–95 %) for an information-absent determination on the same subject’s data. Statistical confidence for all determinations was over 95 %. In other words, all information-present and information-absent subjects’ data were grouped in the correct 5 % range at one end or the other of the distribution. Thus, the spread between the least statistically confident information-present subject’s determination and the least statistically confident information-absent subject’s determination was over 90 percentage points. Statistically, all subjects were extremely far from being misclassified in either a false-negative or false-positive category.

This fact is also reflected in the median statistical confidence of 99.9 % and the mean statistical confidence of 99.5 % for all determinations across all studies using the P300-MERMER.

### Comparison of P300-MERMER-based and P300-based results

Both the analysis using the P300-MERMER and the analysis using the P300 alone produced error rates of 0 %, accuracy rates of 100 %. There were no false negatives and no false positives. There were also no indeterminates.

The median statistical confidence for P300-based determinations was 99.6 %, as compared to 99.9 % for the P300-MERMER. The mean statistical confidence for P300-based determinations was 97.9 %, as compared to 99.5 % for the P300-MERMER.

The statistical confidences for the P300-MERMER-based analysis were significantly higher than those for the P300-based analysis (*p* < .0001, sign test), for the combined data for all studies. P300-MERMER analysis also produced significantly higher statistical confidences (*p* < .0001) in each of the four studies taken separately.

All P300-based determinations were above the pre-established criteria of 90 % for information-present determinations and 70 % for information-absent determinations. All P300-based determinations were in fact above 90 % statistical confidence for both information-present and information-absent subjects. For information-absent subjects, this means that all determinations were at least 20 percentage points above the criterion. Some of the information-present determinations, however, were close to the 90 % criterion. Unlike the P300-MERMER-based analysis, the P300-based analysis resulted in statistical confidences for some subjects’ results that were close to (albeit still above) the threshold between a correct determination and an indeterminate outcome.

All determinations, however, were far from either a false positive or a false negative, even when using only the P300. With the P300-based analysis, all information-present and information-absent subjects’ data were correctly grouped in the correct 10 % range at one end or the other of the distribution, as compared with the 5 % range for the P300-MERMER-based analysis. Thus, for the P300-based analysis, the spread between the least statistically confident information-present subject’s determination and the least statistically confident information-absent subject’s determination was over 80 percentage points. Statistically, all subjects were extremely far from being misclassified in either a false-negative or false-positive category with both the P300-based analysis and the P300-MERMER-based analysis.

In 43 of 76 subjects (57 %), the statistical confidence for the P300-MERMER-based determination was higher than the statistical confidence for the P300-based determination. In these 43 subjects, the P300-MERMER produced a mean increment in statistical confidence of 2.7 % over the P300. For all subjects combined, including those where the statistical confidences were equal for P300-MERMER and P300, the mean increment produced by the P300-MERMER over the P300 was 1.5 %.

The advantage of the P300-MERMER over the P300 may have been limited by a ceiling effect. The statistical confidence for both P300-MERMER-based and P300-based analyses was 99.9 % for 28 of 76 subjects (37 %). Considering only the subjects whose P300-based analysis yielded less than the maximum possible 99.9 % confidence, in 43 of 48 cases (90 %), the P300-MERMER-based analysis yielded a higher statistical confidence than the P300-based analysis.

## Discussion

The four brain fingerprinting studies used two types of brain fingerprinting tests to detect two different types of real-life information. The CIA Real Life Study and the Real Crime Real Consequences $100,000 Reward Study were specific issue tests. They detected specific issue knowledge, that is, knowledge regarding specific crimes (or, in some cases in the CIA study, other real-life events). The FBI Agent Study and the Bomb Maker Study were specific screening tests. They detected specific group knowledge, that is, knowledge known to people with particular training, expertise, or familiarity with the inner workings of a particular group or organization.

These two types of tests address two fundamental needs in law enforcement, criminal justice, counterterrorism, and national security. One is to determine objectively whether or not a specific suspect has information stored in his brain that is known only to participants in a particular crime and to investigators. The other is to determine objectively whether or not a particular suspect has knowledge that is unique to individuals with particular training or expertise, such as IED making, or to members of a particular organization or group, such as Al-Qaeda-trained terrorists or members of a terrorist cell or a foreign intelligence agency. Brain fingerprinting proved highly accurate in detecting both types of real-world information stored in the brains of subjects. It also proved highly accurate in detecting the absence of such information in the brains of subjects who did not possess the relevant information.

### Study 1: the CIA Real Life Study

The purpose of this study was to detect concealed information regarding real-life events, including several major crimes, stored in the brains of individuals who had participated in these events. Brain fingerprinting was highly accurate in detecting the presence or absence of this information. Error rate was 0 %. There were no false negatives and no false positives; there also were no indeterminates. Statistical confidence for the individual determinations was extremely high: the median statistical confidence was 99.9 %. In fact, the results for all but one subject achieved a 99.9 % statistical confidence; one subject’s results were at a 98.6 % statistical confidence.

This study, taken together with the voluminous previous research on the reliability of the P300 (e.g., Fabiani et al. 1987; see Farwell 2012) and previous brain fingerprinting research (Farwell 1992, 2012; Farwell and Donchin 1991; Farwell and Smith 2001), indicates that brain fingerprinting can be accurately and reliably applied in the detection of specific issue information in real-world settings.

This study detected information about real-world events in the lives of subjects. Although some of these were serious crimes, all circumstances were such that there were no substantial consequences for the subjects depending on the outcome of the brain fingerprinting tests. Consequently, the level of motivation of subjects did not approach the level of motivation characteristic of many criminal and counterterrorism investigations. The second study addressed this issue.

### Study 2: the Real Crime Real Consequences $100,000 Reward Study

Like the previous study, the Real Crime Real Consequences $100,000 Reward Study detected information regarding real-life events. The added feature of this study was that all of the events were crimes, and subjects faced life-changing consequences based on the outcome of the brain fingerprinting tests. In some cases brain fingerprinting provided evidence in legal proceedings where subjects faced judicial consequences such as the death penalty or life in prison.

In cases where the legal situation was such that the legal consequences of the outcome of the brain fingerprinting test were not life changing, we introduced the following life-changing feature. Such subjects were offered a $100,000 reward for beating the test, that is, for producing an information-absent determination when they in fact knew the relevant information (as a result of having participated in the crime).

Since in the field it is unknown whether or not subjects have learned countermeasures from some outside source, such perpetrators were also taught countermeasures that have proven to reduce the accuracy of other, non-brain fingerprinting techniques (Rosenfeld et al. 2004, 2008; Mertens and Allen 2008) but not of brain fingerprinting (Farwell 2011a, b, 2012). (Note, however, that as discussed below these alternative techniques were already inaccurate and unreliable enough that they would be unsuitable for application in field conditions, even without countermeasures; see Farwell 2011a, b, 2012.)

Error rate was 0 %; accuracy was 100 %. 100 % of determinations were correct. Determinations were achieved with a very high statistical confidence: median statistical confidence was 99.9 %. All but two subjects’ results achieved 99.9 % confidence, and all subjects’ results achieved at least 99.0 % confidence.

Countermeasures had no effect on the outcome of the brain fingerprinting tests.

The results of the Real Crime Real Consequences $100,000 Reward Study show that brain fingerprinting is highly accurate and reliable in field conditions, with the extreme motivations, uncertainties, possible countermeasures, and other complications inherent therein.

Due to the high consequences of the outcome of forensic tests in field applications, it is important to guard against false positives by establishing a high criterion for an information-present response. It is highly valuable and desirable to obtain results that are well above even this high criterion. When subjects were facing the death sentence or life in prison, and the outcome of the brain fingerprinting test had a bearing on the relevant legal proceedings, the statistical confidence of our correct information-present or information-absent result was in every case at least 99.9 %.

Studies 1 and 2 were specific issue studies that detected specific issue knowledge, that is, knowledge of specific crimes or other events. Brain fingerprinting is also applied in detecting another type of real-world knowledge, as illustrated by Studies 3 and 4.

### Study 3: the FBI Agent Study

The FBI Agent Study and the Bomb Maker Study applied brain fingerprinting in detecting real-world specific group knowledge, that is, knowledge that is known to people with a particular type of training, expertise, or inside involvement with a specific organization or group.

The FBI Agent Study demonstrated that brain fingerprinting reliably and accurately detects information known to people with specific training and membership in a particular specialized organization, specifically FBI agents. The purpose of this study was to test the ability of brain fingerprinting to detect information stored in the brains of members of terrorist organizations, foreign intelligence agencies, organized crime groups, etc.

Brain fingerprinting detected the relevant knowledge in the brains of FBI agents correctly in 100 % of cases, and with a very high statistical confidence in every case. There were no false negatives, no false positives, and also no indeterminates. The median statistical confidence was 99.9 %.

Unlike the subjects with the specific issue knowledge detected in Studies 1 and 2, the individuals who possess the information detected in the FBI Agent study did not participate in a particular event at a particular time and place. Rather, they obtained essentially the same body of knowledge over a period of time at different times and places in their lives in the real world. The results of the FBI Agent Study demonstrate that brain fingerprinting is accurate and reliable in detecting such knowledge.

### Study 4: the Bomb Maker Study

The Bomb Maker Study, like the FBI agent study, used brain fingerprinting in a specific screening application to detect specific group knowledge. In this case, the knowledge detected was directly relevant to the current national security situation. In current operations, improvised explosive devices (IEDs) account for a high percentage of casualties both to military personnel and to civilians. The terrorists who make and plant the bombs are often very difficult to distinguish from innocent civilians. All bomb makers do, however, have one fundamental defining characteristic. All of them have knowledge and expertise regarding explosive devices stored in their brains.

The Bomb Maker Study used brain fingerprinting to detect knowledge regarding IEDs and explosive ordnance disposal (EOD). Information-present subjects were experts in the field. Information-absent subjects were not.

Brain fingerprinting detected the presence and absence of bomb-making knowledge with a 0 % error rate, 100 % accuracy. There were no false negatives, no false positives; there were also no indeterminates. The median statistical confidence for P300-MERMER-based determinations was 99.9 %. The results show a very high level of statistical confidence that all of the brain fingerprinting results are not only correct, but are very far statistically from either a false negative or a false positive.

The present study achieved a level of accuracy and statistical confidence more than adequate for field use in detecting knowledge specific to bomb makers or detecting other specialized knowledge or expertise. Moreover, we believe that the accuracy of this test can be improved further by increasing the number of trials so as to increase the signal-to-noise ratio.

In light of the high percentage of casualties currently caused by improvised explosive devices (IEDs), the Bomb Maker Study demonstrates one area where brain fingerprinting addresses a major current need in national security.

### Do these methods produce sufficiently low error rates and sufficiently high statistical confidences for field application?

In our view, in order to be viable for field use, a technique must produce less than 1 % error rate overall and less than 5 % error rate in every individual study. Applying the methods and meeting the scientific standards described herein in these four studies produced results that far exceed this standard. Error rate was less than 1 % overall, and less than 1 % in each individual study. This is also the case with our previous studies, and with independent replications of these same methods elsewhere as described above. Although we describe the results as “less than 1 % error rate,” these specific methods have in fact never produced a single error, neither a false positive nor a false negative, in our laboratory or elsewhere (e.g., Allen and Iacono 1997). In our view this level of error rate is viable for field use. Moreover, the countermeasures that have dramatically reduced the accuracy of other techniques have had no effect on the methods we report here and in our previous studies, as described herein.

Regarding statistical confidence, we suggest a change in criteria based on these field results. Our a priori criterion for an information-present determination was 90 % statistical confidence. Our a priori criterion for an information-absent determination was 70 % statistical confidence in the opposite direction. These were set before the data were collected. Even with a criterion of 95 % statistical confidence for both information-present and information-absent determinations, error rate for all four studies would be 0 %. The determinations computed according to these methods would have been 100 % accurate, with no false positives and no false negatives. There also would still have been no indeterminates with the P300-MERMER based analysis.

In view of our results, we suggest that a higher criterion for both information-present and information-absent determinations is in order for future field applications of these methods. A criterion of 95 % statistical confidence for an information-present determination, and 95 % confidence in the opposite direction for an information-absent determination, would in our view be appropriate.

Our results suggest that following these specific methods and meeting these specific scientific standards provides sufficient conditions for viable field use of brain fingerprinting technology in detecting concealed information in real-world situations where the outcome of the test may result in substantial consequences. In our view, it would be a serious error, and in some cases a serious violation of rights, to fail to apply or at least to make available for voluntary use this technology to determine the truth in cases where not knowing the truth can have serious consequences. Detecting or failing to detect the truth regarding what information certain individuals possess or do not possess is often critical to solving a crime or addressing a threat to national or global security. In our view, brain fingerprinting, when practiced and interpreted according to the scientific standards outlined herein, can be a valid, accurate, and reliable means to discover such truth.

### Future implications of the difference in P300-MERMER and P300 results

In all four studies, the analysis using the P300-MERMER produced significantly higher statistical confidences than the analysis using the P300 alone. Error rates were the same for both analysis methods. The identical error rates may have been due to a ceiling effect, given that error rates were 0 % (100 % accuracy) for both the P300-MERMER and the P300, and both techniques produced results that were extremely far from an error in every case. No technique is 100 % accurate forever, however. In the future the higher statistical confidences provided by the P300-MERMER will be likely to result in a lower error rate as well, when and if eventually the technique produces errors.

Also, the P300-MERMER analysis yielded results that were quite far from an indeterminate, whereas the P300 yielded results for a few subjects that were close to an indeterminate outcome. It is likely that in the future the P300 will produce more indeterminates than the P300-MERMER, even when neither technique produces an error or even anything close to an error.

### What methods are necessary and sufficient to obtain less than 1 % error rate and high statistical confidence for every individual determination?

#### Different methods produce different results

The error rates for all four of the current studies were less than 1 %. The median statistical confidence for individual determinations was 99.9 %. Countermeasures had no effect. Previous studies that used the same or very similar methods have achieved very similar results: the same error rate, and nearly as high statistical confidences. These include the original Farwell and Donchin (1991) brain fingerprinting study, Farwell (1992), Farwell and Smith (2001), and the replication of the brain fingerprinting methods by Allen and Iacono (1997). (For a review, see Farwell 2012).

The results achieved when the standards have been followed are dramatically different, however, from the results achieved in studies that have applied different methods in an attempt to obtain the same results. Our data suggest that meeting all of the 20 brain fingerprinting scientific standards specified above is sufficient to produce less than 1 % error rate, extremely high statistical confidence for each determination, a low (or 0) rate of indeterminates, and extremely high resistance to countermeasures. Additional research is required to determine which of these standards are also necessary conditions for such performance.

Farwell and Donchin (1991) met 17 of the 20 standards, all but standards 4, 8, and 10. This demonstrates that these three standards, although part of the set of sufficient standards to produce high accuracy and statistical confidence, are not necessary. These three standards had not been formulated at the time of Farwell and Donchin. Standard 4 was developed in the FBI Agent Study reported herein, as described above. Standards 8 and 10 were developed in response to the challenges of field applications in situations with major consequences of the outcome.

Research in other laboratories suggests that at least the most essential of these standards are necessary for valid, accurate, and reliable results. Several studies that used different methods reported error rates an order of magnitude higher than those of the present study and previous similar ones, as well as much lower statistical confidences (no better than chance for information-absent subjects). (For a review see Farwell 2012). For example, Rosenfeld et al. (2004) reported overall 35 % error rate without countermeasures and 67 % error rate with countermeasures. Rosenfeld et al. (2008) and subsequent studies on the “complex trial protocol” reported an overall error rate of 15 % without countermeasures and 29 % with countermeasures. In all of these studies, statistical confidences (when reported) for information-present subjects were over 90 %, and statistical confidences for information-absent subjects averaged approximately 50 % (chance). Approximately half of the statistical confidences for information-absent subjects were less than chance (50 %). Miyake et al. (1993) reported an error rate of 35 %, with 17 % indeterminate. Mertens and Allen (2008) reported varying error rates for different methods, ranging from 18 % error rate with 58 % indeterminate to 42 % error rate with 0 % indeterminate.

Different methods produce different results. The standard brain fingerprinting methods applied in the current studies have produced the kind of results reported herein, and similar results in independent replications elsewhere (e.g. Allen and Iacono 1997). Different methods have produced dramatically different results—more than an order of magnitude higher error rates, dramatically lower statistical confidences (some methods averaging no better than chance for information-absent subjects), and susceptibility to countermeasures. We suggest that the differences in methods responsible for these differences in results have included the features discussed below. These are discussed in more detail, with specific reference to all related publications to date in English, in Farwell (2012). The following methods are highly valuable in our view for achieving results comparable to those of the present studies. Which standards are entirely necessary is a matter to be clarified by future research.

#### Use crime-relevant targets and plausible irrelevants

This is addressed by brain fingerprinting scientific standards 4–6.

We use targets that are relevant details of the investigated situation that have been revealed to the subject, and irrelevants that are plausible but incorrect situation-relevant details. For an information-present subject, the probes are virtually identical to the targets. The only difference is that a different button is pressed. For an information-absent subject, the probes are indistinguishable from the irrelevants. This allows us to directly address the critical question: For this subject, are the probes, like the targets, known details of the investigated situation, or are the probes, like the irrelevants, unknown or irrelevant but plausible details of the investigated situation. This allows us to use a powerful classification algorithm to answer the corresponding statistical question.^5^

Methods that use fundamentally different types of targets, such as meaningless number strings (Rosenfeld et al. 2008 and subsequent “complex trial protocol” studies), do not allow for a meaningful and accurate comparison of probe and target responses, thus disallowing the use of a classification algorithm and eliminating the possibility of obtaining high or meaningful statistical confidences for information-absent subjects.

#### Use a classification algorithm, not a comparison algorithm

This is addressed by brain fingerprinting scientific standards 14–16.

We apply a classification algorithm to classify the probe responses as being more similar to the target responses, which contain a P300-MERMER, or to the irrelevant responses, which do not. We use bootstrapping on correlations to determine whether the probe responses are more similar to the target responses or to the irrelevant responses. Bootstrapping computes the probability that the probe responses are more similar to the target responses, or to the irrelevant responses. This is the statistical confidence for each determination. If the probability is not high enough in either direction, then no determination is made; the outcome is indeterminate (although there were no indeterminates in the present studies.)

An alternative method is to ignore the target responses and use a comparison algorithm to determine if the probe responses are “larger” (variously defined) than the irrelevant responses (Rosenfeld et al. 2004, 2008; and all other studies from that laboratory). This results in two difficulties. First, according to both the predictions of the statistical model and the actual results reported in these studies, either the statistical confidence will be at or below chance, or the accuracy will be at or below chance. Second, if a reasonable, meaningful criterion (such as 90 %) is established for information-present determinations, then some subjects will be determined as information absent when there is up to an 89 % computed statistical probability that the opposite determination would be correct, and the selected determination is incorrect. The mathematical and statistical reasons for this are as follows.

Bootstrapping on P300 amplitude (however amplitude is defined) computes the probability that the probe P300s are “larger” than the irrelevant P300s. This constitutes the probability that information present is the correct determination, and thus provides a meaningful statistical confidence for an information-present determination (sometimes called “guilty”). All subjects whose data fail to meet the criterion (usually 90 %) for an information-present determination are classified as information absent (or “innocent”). This means that if the probability is as high as 89 % that information-present is the correct classification, the subjects is classified as information absent, with a statistical confidence of 11 %. This results in a determination that places a subject in a category where the statistics computed have determined a probability of up to 89 % that the *opposite* determination is in fact correct, and the selected determination is incorrect.^6^

This situation cannot be corrected by simply changing the criterion. This is because if the true state of the subject is information absent, then the probe and irrelevant waveforms are expected to be identical, and the expected value of any statistic to determine whether the probe responses are “larger” than the irrelevant responses is 50 %. That is, if the statistics work as designed, the average statistical confidence for an information-absent determination will be 50 %. The actual statistical confidences reported in, for example, Meixner et al. (2009) do in fact average approximately 50 % (chance) for information-absent subjects.^7^ If the criterion is lowered so that an information absent classification requires at least a better than chance statistical confidence (>50 %), then the accuracy rate falls to chance (50 %) or less, since on average half of the information-absent subjects will have less than 50 % statistical confidence. This is borne out by both the predictions of the statistical model and the actual reported data for the studies cited above that use bootstrapping to implement the comparison method rather than the classification method.

Rosenfeld et al. (2004, 2008) and their similar studies also demonstrated definitively that their comparison algorithm is much more susceptible to countermeasures than the classification algorithm applied in Farwell and Donchin (1991), Farwell and Smith (2001), and the present studies. To date, no one has ever beaten a brain fingerprinting test with countermeasures, despite a $100,000 reward for doing so. One reason for this difference is that with the comparison algorithm of Rosenfeld et al., all a subject has to accomplish with countermeasures is to reduce the statistical confidence for an information-present determination to less than 90 % (equivalent to greater than 10 % statistical confidence that information-absent is the correct determination), and he will be determined to be information absent.

With a classification algorithm, by contrast, an information absent-determination requires a meaningfully high statistical confidence that the subject is in fact information absent. In the studies reported herein, for example, all statistical confidences were over 95 % using the P300-MERMER and over 90 % using the P300.

For these reasons, the comparison method is inadequate for real-world forensic use. Obviously, there is no practical use in the real world for a result where there is as low as 11 % statistical confidence that the result is correct, as computed by the actual statistics applied to reach that result. It is simply not viable for an expert to testify in court, “We determined the subject to be information absent, and we have an 11 % confidence that this is the correct determination.” Even the average statistical confidence for information-absent determinations with the comparison method—50 %, no better than chance—is obviously insufficient for application in any case with non-trivial consequences.

Various classification methods using bootstrapping, and other classification methods using other statistical techniques, may be adequate for classifying the brain responses. The method we apply in the current research and the other replications cited above, using bootstrapping on double-centered correlations, has several advantages that we are convinced constitute one of the factors leading to the low error rate and high statistical confidence produced by this method. Bootstrapping on correlations provides a precise metric of the similarity between the pairs of response types, probe-target and probe-irrelevant. This metric takes into account not only the amplitude but also the latency, time course, and morphology of the response. It extracts information from every point in the relevant time epoch of the waveform (Farwell 2012).

#### Establish high and meaningful statistical confidence criteria for both information-present and information-absent determinations

This is addressed by brain fingerprinting scientific standard 17.

We compute the probability that the probe response is more similar to the target response, or, alternatively, to the irrelevant response. (The latter probability is 100 % minus the former.) The former is the statistical confidence for an information-present determination. The latter is the statistical confidence for an information-absent determination. We set a criterion for an information-present determination that requires a reasonable level of probability that this determination is correct. In the present study the criterion was 90 %. All information-present determinations would still have been correct, however, if we had used a 95 % criterion.

We set a separate criterion for an information-absent determination that also requires a reasonable level of probability that the determination is correct. In the present studies we used a 70 % criterion for information-absent determinations. All determinations would still have been correct, however, if we had used a 95 % criterion.

If a subject’s data do not meet either criterion, then the subject is not classified as either information present or information absent. The outcome is indeterminate.

When only one criterion is set, then, if the criterion is high enough to be meaningful for information-present determinations, it is not viable for information-absent determinations. As described immediately above, use of only a single criterion inevitably results in substantial decrements in statistical confidence, accuracy, or both.

#### Inform subjects of the significance of probe stimuli

This is addressed by brain fingerprinting scientific standards 7–10.

We describe the significance of each probe stimulus in the context of the investigated situation, without of course revealing to the subject which stimulus is the probe and which are similar irrelevants. For example, “Mr. Subject, you will see a knife, a pistol, a rifle, and a baseball bat. One of these is the murder weapon.”

The P300-MERMER manifests the process of context updating. That is, the subject takes note of relevant, meaningful information delivered by the stimulus. Things are significant to a person in context. Establishing the context of the investigated situation, and the significance of each probe stimulus in that context, ensures that if the subject knows the relevant information he will recognize the probes.

For example, if the victim wore a yellow blouse, simply presenting the words “yellow,” “blue,” “red,” and “green” as stimuli, without any explanation of the context in which one of these is meaningful, would likely be ineffective. Telling the subject that one of the stimuli would be an item of clothing worn by the victim, and then presenting “yellow blouse,” “blue skirt,” etc., would be much more likely to elicit a response revealing the concealed knowledge for a subject who possessed it.

It is not surprising that studies that did not describe the significance of the probes have reported higher error rates. This is discussed in detail in Farwell (2012).

#### Confine conclusions to what the science actually measures

This is addressed by brain fingerprinting scientific standard 18.

Brain fingerprinting detects information stored in the brain. It does not detect how it got there. It does not detect what a subject *should* know, *would* know, or *could* know and under what circumstances (e.g., if he committed a crime). It only detects what a subject *actually does know*. It detects information. It does not detect guilt or innocence. It does not detect past actions.

Science, and the scientific testimony of expert witnesses in cases where brain fingerprinting is applied, must confine conclusions to what the science actually shows. A brain fingerprinting test shows that the subject does or does not know the specific details about the investigated situation embodied in the probe stimuli. It is up to the attorneys to argue, and the judge and jury to decide, based on brain fingerprinting and all other available evidence, whether a crime took place, what the crime was, and who is guilty or not. When brain fingerprinting experts have testified in court (e.g., Harrington v. State 2001), they have correctly confined their testimony to explaining the science and presenting the result in terms of what the subject knows or does not know.

Prior to a brain fingerprinting test, a criminal investigator investigates the crime, develops his theory of the crime, and develops a set of relevant information embodied in the probe stimuli that he believes constitute the salient features of the crime. This is the non-scientific opinion of the criminal investigator, based on his professional expertise and judgment. The scientist who testifies regarding the brain fingerprinting test does not opine on whether the suspect took part in a crime. He does not even opine on whether the information contained in the probe stimuli which was provided by the criminal investigator accurately represents the crime, or whether a crime took place at all. His testimony about the case is confined to the scientific conclusion that is warranted by the data: at the time of the test, this subject does or does not know these specific details about the crime. These results must be interpreted in light of all of the other available evidence.

Brain fingerprinting provides objective evidence of the contents of human memory. Witness testimony provides a subjective (and not always truthful) account of the contents of human memory. Like eyewitness testimony, brain fingerprinting evidence must be evaluated in light of the well-known limitations of human memory. Judges and juries must apply the same considerations in evaluating brain fingerprinting evidence that they do in evaluating witness testimony. All of this is in the domain of the judge and jury. The role of the brain fingerprinting scientist is simply to present the scientifically established facts regarding what the subject does or does not know, along with an explanation of the science that establishes these fact.

Some commentators, and even some researchers, have attempted to draw unwarranted conclusions that go far beyond what the science actually shows, far outside the realm of science and into the rightful domain of the judge and jury. Both critics and supporters have made the error of maintaining that brain fingerprinting detects guilt, innocence, or past actions, rather than simply detecting information. Some have opined that brain fingerprinting *should* detect guilt, or lying, or participation in past actions, or a variety of other things that it does not detect, and have found fault with brain fingerprinting because of the various things it does not detect. In our view, brain fingerprinting detects what it detects—presence or absence of information stored in the brain—and there is no reason to hold that it should detect something else.

In any case, any legitimate scientific report must be confined to reporting what brain fingerprinting actually detects: the subject’s knowledge or lack of knowledge of the specific information contained in the probe stimuli as relevant in the context of the crime.

Brain fingerprinting is like all other forensic sciences in this regard. A DNA scientist reports that Sample A (ostensibly from the crime scene) matches Sample B (ostensibly from the suspect). He does not conclude that therefore the subject committed a murder. Similarly, brain fingerprinting experts whose testimony as expert witnesses has been ruled admissible in court have confined their testimony to the science, specifically to the finding of what information about the crime the subject possessed, along with an explanation of the scientific principles and methods involved in testing for the presence of that information (Harrington v. State 2001; Farwell 2012).

Some studies that have failed to confine conclusions to what the science measures have consequently reported high error rates. Some examples are described immediately below.

#### Establish adequate experimental control and determine ground truth

This is addressed by brain fingerprinting scientific standards 3, 4, and 18–20.

Experimental control is vital to the validity and success of every experiment. Understanding what the science measures and does not measure is critical for establishing and maintaining proper experimental control.

In the field, the relevant knowledge embodied in the probes is provided to the scientist by the criminal investigator. In the laboratory, the relevant knowledge is fabricated by the experimenter and imparted to the subject through a knowledge-imparting procedure such as a mock crime or training program.

The brainwave test does not test whether or not the subject participated in the crime (in a field study) or the knowledge-imparting procedure (in a laboratory study). Drawing conclusions beyond what the science addresses, and mistakenly assuming that the testing procedure detects (or somehow should detect) past actions rather than knowledge, has led to errors in research that have produced marked decrements in accuracy as well as erroneous conclusions and misinterpretation of results. The mistaken assumption that brainwave testing detects (or should detect) actions rather than knowledge has led some researchers to confound the knowledge-imparting procedure with the knowledge-detection procedure. This may result in apparent low accuracy that is actually caused by the failure of the knowledge-imparting procedure rather than the failure of the knowledge-detection procedure. Some researchers (e.g., Mertens and Allen 2008) implement a knowledge-imparting procedure such as a mock crime, and then test for knowledge that is intended to be imparted by the knowledge-imparting procedure, without any independent verification that the knowledge-imparting procedure actually succeeded in imparting the relevant knowledge. When the knowledge-detection procedure does not detect the knowledge, they conclude that the knowledge-detection procedure failed. In fact, the knowledge-imparting procedure may have failed to impart the knowledge, and the knowledge detection procedure then correctly determined that the knowledge was not there. Failure of the knowledge-imparting procedure to impart the knowledge must not be confounded with failure of the knowledge-detection procedure to detect it. No technique, no matter how perfect, can detect knowledge that is not there to be detected. (For a review and discussion, see Farwell 2012).

One of the reasons that the present studies were able to achieve, correctly evaluate and quantify, and report a high level of accuracy is that the experimental design independently verified the presence or absence of the knowledge that the test sought to detect. In other words, ground truth was properly defined and clearly determined. This was accomplished through post-test interviews and other means, as described above.

#### Require subjects to perform the necessary mental tasks to elicit the telltale brain responses and to prove behaviorally that they have done so on every trial

This is addressed by brain fingerprinting scientific standard 11.

In order to emit a P300-MERMER, the subject must read and process the eliciting probe stimulus. Accommodating subjects such as undergraduate students who are facing no life-changing consequences of the outcome of the test can generally be trusted to read and process the stimuli simply because they are instructed to do so. Subjects in field tests whose life or freedom may depend on the outcome of the test are less accommodating. In our field experience, such subjects will do everything they can to avoid cooperating with the test, to the extent that they can do so without any overt indication.

In our tests, we require a subject to make a differential button press in response to every stimulus. The subject must read and process the stimulus, determine whether or not it is a target, and press the appropriate button on every trial. The button-press task *requires the subject to read and process every stimulus and to prove that he has done so on every trial*.

In some alternative techniques such as the “complex trial protocol” (Rosenfeld et al. 2008 and subsequent studies), there is no such requirement. Stimuli alternate one-to-one, totally predictably, between a target or nontarget (meaningless number strings) followed by a meaningful probe or irrelevant stimulus. Differential button presses are required on every other stimulus presentation, only to the target/nontarget stimuli. In response to the probe and irrelevant stimuli, the subjects is only required to press an “I saw it” button indicating that something appeared in that general area of the screen. In the predictably alternating sequence, the subject can read and process the target/nontarget stimulus and make the appropriate button press, then ignore or not even look at the next probe/irrelevant stimulus but simply press a button when there is a brightening in that area of the visual field, and then read and respond to the next target/nontarget, and so on.

By not reading and processing the probe and irrelevant stimuli, the subject can perform the necessary task on every trial and avoid emitting a differential brain response indicating that he has recognized the situation-relevant probes. The analysis compares the probe and irrelevant responses as described above in the discussion of comparison versus classification algorithms. (Targets and nontargets are ignored in the analysis.) The subject is instructed to read and process the probe and irrelevant stimuli, but the task demands do not require him to do so and to prove that he has done so on every trial.

Motivated subjects with something real to hide and non-trivial consequences of being detected do not read and process the probe and irrelevant stimuli (Farwell 2012). They can and do perform the overt task as instructed without exhibiting any brain responses revealing their concealed knowledge. We verified this in the course of the present research.

In the present research program, we began a fourth study on the “complex trial protocol” that we discontinued for scientific and ethical reasons. We applied this method to test information-present subjects who had already been correctly detected with over 99 % statistical confidence by brain fingerprinting. The “complex trial protocol” produced a 100 % error rate, 0 % accuracy, for our first three motivated, real-world subjects. This is because all such subjects whom we tested figured out on their own how to beat the test. The simple method they used is not a countermeasure that must be taught, but simply the way that motivated subjects with something real to hide respond to the task requirements, as described immediately above. (In the absence of non-trivial consequences, accommodating laboratory subjects do not respond in this way, and are detected with greater than chance accuracy.) We discontinued the study when we concluded that it would be unethical to administer the “complex trial protocol” to any more subjects in a field setting with real-world consequences, in light of the 0 % accuracy rate and other shortcomings described above.

It has been suggested (Rosenfeld et al. 2008) that subjects can be coerced or tricked into reading and processing the probe and irrelevant stimuli by periodically asking the subject to remember what stimuli have been presented, and threatening to file a false report that the subject has “failed” the brainwave test if he does not correctly remember the stimuli. Such a report would be patently false, and in the real world the threat to file such a false report would be empty, because anyone who knows how the test works will realize that such a subject has in fact passed the brainwave test: his brain responses will show no indication of his possessing the concealed knowledge. In our view, such false threats are unethical in real-world cases.^8^ In any case, such threats are ineffective with a knowledgeable subject who has something real to hide and is facing non-trivial consequences. (For a detailed discussion, see Farwell 2012.)

#### Present a sufficient number of trials for adequate signal-to-noise enhancement, and use effective signal-processing techniques

This is addressed by brain fingerprinting scientific standard 13. All or virtually all studies that have reported low accuracy rates have failed to meet this standard (see Farwell 2012 for a review).

While the brain is engaging in the information-processing tasks of interest in our experiments, it is also engaging in a host of other activities. Consequently the signals that we seek to detect are mixed with myriad other patterns in the EEG, which for our purposes are noise. Fortunately, however, the signals of interest are time locked to the stimulus, since they manifest information-processing brain activity elicited by the stimulus. The other brain activity is not time locked to the stimulus. When the stimulus is presented many times and the responses time locked to the stimulus are averaged, the noise, which is not time-locked to the stimulus, averages to zero. Thus, noise is eliminated or minimized, leaving the signal that is of interest for our purposes. The more trials we include in the average, the greater the reduction of noise, the greater the signal-to-noise enhancement, and the greater the accuracy and statistical confidence of the results.

Bootstrapping, through repeated iterative sampling and averaging, provides a means of creating smooth, relatively noise-free averages while reintroducing the variability across the single trials that contribute to the averages. Accounting for this variability is essential for establishing the statistical confidence of the determination, and creating a smooth average is essential for applying the classification algorithm. We have found that using a minimum of 100 probe trials with a ratio of 1/6 probes, 1/6 targets and 2/3 irrelevants produces excellent results. Presenting fewer than 100 probe trials may produce adequate results, but in some studies has produced markedly lower accuracy (see Farwell 2012).

We also applied other signal-processing refinements that may have increased the accuracy and statistical confidence of the results obtained. For example, we used equal-ripple, optimal, finite impulse response, digital filters that are optimal in the strict mathematical meaning of the term. These have been shown to be highly effective in reducing noise while maintaining the amplitude, latency, and morphology of the response (Farwell et al. 1993). Such signal-to-noise enhancement techniques may have contributed to the accuracy we obtained, particularly since our classification algorithm takes into account all points in the relevant time epoch of the waveform.

## Summary

The purpose of this research was as follows: (1) To test our specific brain fingerprinting methods in a variety of field applications, including situations with major life-changing consequences such as the outcome of a criminal trial or a $100,000 reward for beating the test; (2) to compare the performance of the P300-MERMER and the P300 with respect to error rate and statistical confidence; (3) to identify the scientific standards that are sufficient to produce low enough error rates, high enough statistical confidences, and high enough resistance to countermeasures for real-world field applications; and (4) to examine which of these standards may be necessary to produce such results, by identifying the differences in methods that have produced markedly different results in other published research.

We applied brain fingerprinting in two types of tests: (1) specific issue tests to detect knowledge of a specific event, including felony crimes with real-world consequences of the outcome; and (2) specific screening or focused screening tests to detect knowledge characteristic of people with specific training, expertise, or inside information of a specific organization or group.

Two specific issue studies detected concealed information regarding (1) real-life events including felony crimes; (2) real crimes with substantial consequences (either a judicial outcome, i.e., evidence admitted in court, or a $100,000 reward for beating the test).

Two specific screening studies detected concealed information regarding (1) knowledge unique to FBI agents; and (2) knowledge unique to explosives (EOD/IED) experts.

We analyzed the data for each subject twice. First we analyzed the data using the using only the P300 (a positive peak). Then we analyzed the same data using the full P300-MERMER, inclusive of both the positive P300 peak and the subsequent negative peak (late negative potential or LNP). We compared the results of these two analysis methods in terms of error rate and statistical confidence. With both P300 and P300-MERMER, error rate was 0 %: determinations were 100 % accurate, i.e., there were no false negatives and no false positives. Also, there were no indeterminates.

In all four studies, statistical confidences were very high with both the P300-MERMER and the P300 alone. Median statistical confidence for determinations was 99.9 % with the P300-MERMER and 99.6 % with the P300 alone. Mean statistical confidence was 99.5 % with the P300-MERMER and 97.9 % with the P300.

All determinations with the P300-MERMER, both information-present and information-absent, achieved over 95 % statistical confidence. In studies 1 and 2, the specific issue studies, all determinations with the P300-MERMER achieved at least 99 % confidence.

The brain fingerprinting data-analysis algorithm using the P300 alone produced the same determinations: 100 % of determinations were correct. Error rate was 0 %. Accuracy was 100 %. There were no false negatives and no false positives. Also, there were no indeterminates.

For most of the subjects (57 %), the statistical confidence with the P300-MERMER-based analysis was higher than with the P300 alone. In the remaining subjects the two statistical confidences were the same. The statistical confidences for the P300-MERMER were significantly higher than for the P300 (*p* < .0001) in all studies taken together, and also in each individual study. All determinations with the P300, both information-present and information-absent, achieved over 90 % statistical confidence.

We taught subjects the same countermeasures that had substantially decreased the accuracy and statistical confidence of other, alternative techniques, and offered them a $100,000 reward for beating the test. Countermeasures had no effect on our technique.

The present study, our previous publications, and independent replications in other laboratories have produced markedly different results from the fundamentally different methods of some laboratories that have published related research. Some alternative methods have produced substantially different results that are not viable for field applications.

We have identified the specific methods, in particular the 20 scientific standards, that appear to be sufficient to produce the results that we have obtained in this and previous studies, and the similar results that others have obtained in independent replications of our methods. Our research has focused on identifying the sufficient conditions for obtaining results that are viable for field applications.

Several other laboratories have contributed substantially to identifying which of these standards are also necessary for obtaining such results. Studies that have applied substantially different methods have produced error rates 10–50 times higher than those of our studies (e.g., Mertens and Allen 2008; Rosenfeld et al. 2004, 2008). They have also produced dramatically lower statistical confidences. In two series of studies conducted by another laboratory, failure to meet certain specific standards has consistently resulted in statistical confidences averaging no better than chance for information-absent subjects (Rosenfeld et al. 2004, 2008). Failure to meet the scientific standards outlined herein has also resulted in susceptibility to countermeasures in several studies applying alternative methods (Mertens and Allen 2008; Rosenfeld et al. 2004, 2008).

In our view, in order to be viable for field use, a technique for detection of concealed information with brainwaves must produce less than 1 % error rate overall, and less than 5 % error rate in each individual study. It must consistently produce these results in situations with major, life-changing, real-world consequences. It must be extremely resistant to countermeasures, and must produce these results in real-world situations, where it is unknown whether the subject is practicing countermeasures or not.

There can be some flexibility in statistical confidence criteria, as long as the statistical confidence is reported for each determination, both information-present and information-absent. In light of our results, however, we suggest for the future field applications a statistical confidence criterion of at least 90 % and preferably 95 % for information-present determinations, and at least 90 % and preferably 95 % in the opposite direction for information-absent determinations. The results of these studies, along with our previous studies and independent replications in other laboratories, suggest that following the methods and scientific standards outlined herein provides the sufficient conditions to achieve such a level of error rate and statistical confidence.

In our view, these results, along with our previous publications and independent replications of our methods in other laboratories, suggest that the methods applied herein provide a level of error rate, statistical confidence, and resistance to countermeasures appropriate for field applications. In our view, the decision to rule brain fingerprinting admissible in court in the United States was well founded in science, with the following caveats. To provide valid and reliable scientific evidence, it is necessary that the testing be conducted and interpreted according to the methods and the 20 scientific standards described herein. It is also necessary that scientific testimony of expert witnesses on brain fingerprinting continue to deal exclusively with reporting the specific facts actually demonstrated by the test—i.e., presence or absence of certain specific knowledge—along with an explanation of the scientific principles and methods that have revealed this result. In our view, the continuing application of brain fingerprinting to discover the truth regarding what information subjects possess about a crime or other real-world field situation serves the interest of justice, and is in the public interest in any jurisdiction where there is a high correlation between truth and justice.

## Figures and Tables

**Fig. 9:**
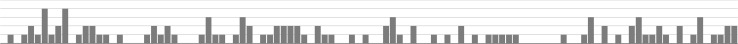
Example of random sampling of 108 probe trials for one bootstrapping iteration, Study 1, subject 1

**Fig. 10:**
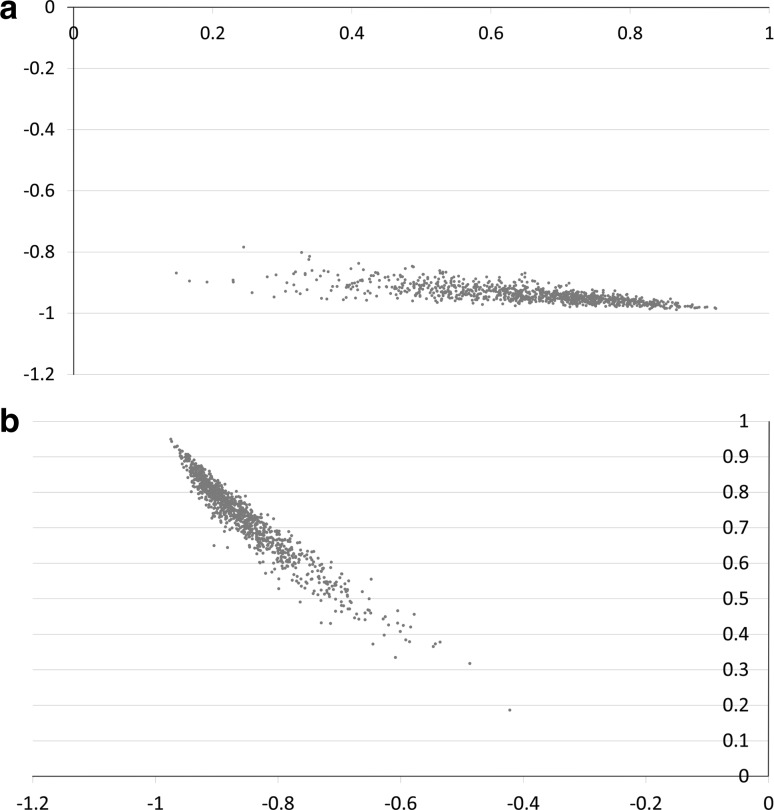
**a** Distribution of correlations for an information-present subject (Study 2, subject 1). X-axis: probe-target correlations. Y-axis: probe-irrelevant correlations. **b** Distribution of correlations for an information-absent subject (Study 2, subject 13). X-axis: probe-target correlations. Y-axis: probe-irrelevant correlations

**Fig. 11:**
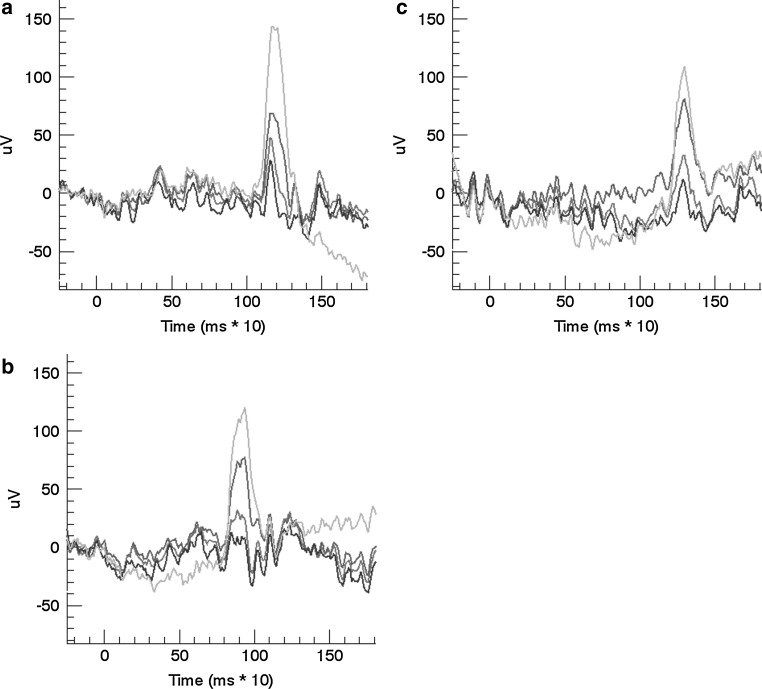
Examples of single trials rejected for artifacts. Brain response at Fz (*red*), Cz (*green*), and Pz (*blue*), plus eye-movement response (EOG—*yellow*)

## Appendix 1: The bootstrapping statistical procedure

Brain fingerprinting computes a determination of “information present” or “information absent” for each subject, along with a statistical confidence for each individual determination. To be valid, the statistical confidence for an individual determination must take into account the level of variability in the individual brain responses that are aggregated in the average response. Farwell and Donchin (1988b, 1991) and their colleagues Wasserman and Bockenholt (1989) applied bootstrapping to compute a statistical confidence for each individual determination that takes this variability into account. These publications describe bootstrapping in detail. It has now become a standard procedure in event-related brain potential research. Bootstrapping is a method to compute probability and statistical confidence regardless of the shape of the distribution of the data. It also provides a means to re-introduce the variability across single trials present in the original data, while preserving the feature of a smooth average that is necessary for comparing the waveforms of the three types.

The algorithm is as follows. Conduct the following procedure twice, once using the time epoch characteristic of the P300-MERMER (typically 300–1,800 ms after the stimulus) and once using the time epoch characteristic of the P300 alone (typically 300–900 ms post-stimulus).

1. Sample randomly with replacement T target trials, P probe trials, and I irrelevant trials, where T, P, and I are equal to the total number of artifact-free trials in the data set of the respective types. A trial consists of one stimulus presentation and the associated brain response data.
2. Average the trials by trial type, yielding three average waveforms: probe, target, and irrelevant. Compare the average waveforms to determine if the probe average is more similar to the target average or to the irrelevant average.
3. Repeat the above procedure 1,000 times. Each iteration yields a new set of 3 average waveforms containing probe, target, and irrelevant trials respectively. Keep a tally of the number of times the probe average waveform is more similar to the target average waveform than to the irrelevant average.
4. For each iteration, compare the probe, target, and irrelevant waveforms according to the following algorithm: (a) subtract the grand mean of all trials, or grand average waveform, from each of the 3 averages, yielding 3 grand-mean-adjusted averages; (b) compute the correlation between the adjusted probe average and the adjusted irrelevant average; (c) compute the correlation between the adjusted probe average and the adjusted target average; (d) compare the probe-irrelevant correlation with the probe-target correlation; (e) if the probe-target correlation is greater, then increment the “information present” tally by one; otherwise, increment the “information absent” tally by one.
5. Compute the percentage of times that the probe-target correlation is greater than the probe-irrelevant correlation. This the percentage of times that the probe waveform is more similar to the target waveform than to the irrelevant waveform. This provides the probability or statistical confidence for an “information present” result. 100 % minus this figure is the probability that the probe response is more similar to the irrelevant response, which provides the statistical confidence that for an “information absent” result.
6. Compare the computed statistical confidence to a decision criterion. If the statistical confidence for an information-present result is greater than 90 %, classify subject as information present. If the statistical confidence for an information-absent response is greater than 70 %, classify the subject as information absent. If neither criterion is met, no determination is made: the subject is not classified as either information present or information absent. This is an “indeterminate” outcome.

If the bootstrapping procedure produces a high statistical confidence (defined by the pre-established criterion, e.g., 90 %) that the probe response is more similar to the target response than to the irrelevant response, then the determination is “information present.” If the bootstrapping procedure produces a high statistical confidence that the probe response is more similar to the irrelevant response, then the determination is “information absent.”

If neither the statistical confidence for “information present” nor the confidence for “information absent” is high enough to meet established criteria, the subject is not classified in either category, and the result is “indeterminate.” Typically a confidence of 90 % is required for an “information present” determination. A lower criterion, typically 70 %, is generally required for an “information absent” determination.

The outcome of brain fingerprinting data analysis consists of two determinations, each of the form “information present/absent, x % confidence,” e.g., “information present, 99.9 % confidence.” One determination is computed using the full P300-MERMER, and one using the P300 alone. This allows us to report one result with the method that applies the most fully established science and is most certain to meet the standard of general acceptance in the scientific community, and one with the method that applies the state of the art and generally produces the highest statistical confidence.

By computing bootstrapped correlation as described above, the brain fingerprinting data analysis algorithm automatically takes into account the amplitude, latency, and morphology (shape and time course) of the brain response. This maximizes the information extracted from the data and also controls for individual differences in brain responses from different subjects.

## Appendix 2: Illustrative graphs of the bootstrapping procedure

Several features of the bootstrapping procedure are briefly illustrated in Figs. 9 and 10.

The first step of bootstrapping is random sampling, as follows: Sample randomly with replacement T target trials, P probe trials, and I irrelevant trials, where T, P, and I are equal to the total number of artifact-free trials in the data set of the respective types. A trial consists of one stimulus presentation and the associated brain-response data.

Figure 9 illustrates this random sampling procedure. This figure displays the probe trials randomly selected in one iteration of the bootstrapping procedure for subject 1 in Study 1. As can be seen in the figure, all trials were selected between 0 and 4 times, inclusive. Each of the 1,000 iterations produced a new random selection of 108 probe trials, and, similarly, a random selection of target trials and one of irrelevant trials. The trials selected in each iteration for each trial type were averaged, and the probe-target and probe-irrelevant correlations were computed for each iteration on these randomly sampled averages.

Figure 10 a illustrates the distribution of correlations for a typical information-present subject (Study 2, subject 1).

Figure 10b illustrates the distribution of correlations for a typical information-absent subject (Study 2, subject 13).

## Appendix 3: Artifact rejection

Trials contaminated by artifacts generated by eye movements or other muscle-generated noise were rejected on-line, and additional trials were presented so that the required number of artifact-free trials was obtained for each block. The criterion for artifact rejection was as follows: trials with a range of greater than 97.7 μV in the EOG channel were rejected.

Figure 11a–c illustrate trials that were rejected for artifacts. These figures display three EEG channels: Fz (red), Cz (green) and Pz (blue), plus one EOG channel (yellow) measuring eye movement-generated electrical activity. In each of these trials, an eyeblink caused a large positive deflection in the EOG channel. As can be seen in the figures, this also caused a similar but smaller positive deflection in each of the EEG channels. The largest artifact is at the Fz channel, which is closest to the eyes. The artifact is of medium size at Cz, and is smallest at Pz. Note that the range in the EOG channel exceeds the criterion of 97.7 μV in each of these trials. Hence each of these trials was rejected, and they were not included in the analysis.

## Footnotes

1 Note that brain fingerprinting is not applicable for general screening, where the experimenter does not know what specific information is being sought, but only for specific screening, where the subject is tested for a particular body of knowledge, training, or expertise that is known to the experimenter.

2 Farwell and Donchin 1991 met 17 of the 20 standards, as discussed below.

3 Two individuals were each a subject in two different tests, one information present and one information absent.

4 Note that indeterminates are not errors, neither false positives nor false negatives. False positives and false negatives can provide flawed evidence to the detriment of the judicial process. An indeterminate provides no evidence in either direction. Correctly reporting accuracy as 100 % minus the error rate allows the reader to compute the true error rate. Reports of “accuracy” that confound indeterminates with false positives and false negatives hide the true error rate. This makes it impossible to compare results with studies that reveal the actual error rates, or to estimate the probability of an error in the results presented in a case. Such practices are incompatible with application in the criminal justice system.

5 This can be done even if the targets are not crime-relevant, as long as they are reasonably comparable to the probes along dimensions that will affect the amplitude and latency of the response (Farwell and Donchin 1991).

6 For example, in Meixner et al. (2009, p. 215, Table 2, “innocent” subject 11) the subject was determined to be ‘‘innocent’’ when the computed probability was 85% that ‘‘guilty’’ was the correct determination (i.e., that the probe P300 was larger than the irrelevant P300, which is the definition of “guilty” in the “Iall” condition). Statistical confidence for this (correct) determination was 15%, far less than chance.

7 For example, in Meixner et al. (2009, p. 215, Table 2) the average probability computed that probe P300s were larger than irrelevant P300s (i.e., the probability that “guilty” was the correct determination) for factually “innocent” subjects was 59 %. This includes two incorrectly determined as “guilty” because the probability was over 90 % that “guilty” was correct. Including only those correctly determined as “innocent,” the average probability that the probe P300 was larger (i.e. that “guilty” was the correct determination) was 49 %, the equivalent of an average 51 % statistical confidence for correct “innocent” determinations, and about half the “innocent” determinations were made with a statistical confidence of less than chance. If the criterion is changed to implement a requirement that subjects be classified as “innocent” with at least a better than chance (50 %) statistical confidence, the error rate would be 67 % for these reported data. This is an accuracy rate of 33 %, considerably less than chance.

8 With accommodating laboratory subjects in the absence of substantial consequences, such threats are unnecessary. Such subjects simply do as they are told, and read and process all the stimuli. Thus, laboratory subjects in artificial situations can be detected with greater than chance accuracy by this method. Nor is it unethical, in our view, to make such threats in a laboratory study, as long as the situation is totally artificial, the threats have no real-world consequences, and there are no non-trivial consequences of the outcome of the test.
